# Multi-Objective Optimization of FDM Dimensional Accuracy for PLA/TPU Blends Based on Entropy Weight-TOPSIS and Orthogonal Array Design

**DOI:** 10.3390/polym18161958

**Published:** 2026-08-10

**Authors:** Pei Li, Tianlu Wei, Li Yang, Jing Zhao, Shuo Wang

**Affiliations:** 1 School of Mechanical and Vehicle Engineering, Bengbu University, Bengbu 233000, China; yang1155@bbc.edu.cn (L.Y.); zhaojingzaozao@163.com (J.Z.); swang2025@bbc.edu.cn (S.W.); 2 Anhui Province Additive Manufacturing Engineering Research Center, Bengbu 233000, China

**Keywords:** fused deposition modeling, PLA/TPU polymer blends, dimensional accuracy, entropy weight–TOPSIS, multi-objective optimization, process–structure–property relationship

## Abstract

Fused deposition modeling (FDM) of polylactic acid (PLA) is plagued by dimensional inaccuracies—thermal shrinkage, warpage, and geometric distortion—that restrict its application in high-precision manufacturing. Blending thermoplastic polyurethane (TPU) with PLA enhances toughness, yet the coupled effects of blend ratio and printing parameters on dimensional accuracy remain unclear. This study establishes a multi-objective optimization framework integrating single-factor experiments, orthogonal design, ANOVA, and entropy weight–TOPSIS. Single-factor experiments combined with TOPSIS first identify the optimal PLA/TPU blend ratio, and orthogonal experiments are subsequently conducted on this optimal ratio to determine the best parameter combination. The 70:30 PLA/TPU blend delivers the optimal comprehensive performance (TOPSIS closeness: 0.680), attributed to favorable phase compatibility and robust interfacial adhesion as verified by XRD and SEM. Range analysis and ANOVA on the orthogonal results identify infill rate and layer height as the dominant factors governing dimensional accuracy (*p* < 0.05). Under the optimized parameter set (60 mm/s, 210 °C, 90% infill, 0.1 mm layer), the total dimensional deviation reaches only 0.256 mm, substantially lower than single-objective counterparts (0.321–0.418 mm). This work offers a validated strategy for precision control in FDM through synergistic optimization of blend formulation and processing parameters.

## 1. Introduction

Additive manufacturing (AM) is revolutionizing traditional production paradigms. Among its various techniques, fused deposition modeling (FDM) has attracted considerable attention due to its cost-effectiveness, operational simplicity, and design flexibility, showing great potential in aerospace, automotive lightweighting, biomedical devices, and high-end consumer products [1,2,3].

Polylactic acid (PLA), an environmentally benign biodegradable polyester, is the most widely used FDM filament owing to its excellent mechanical properties, favorable processability, and biodegradability, aligning with global “dual carbon” strategies and sustainable manufacturing [4]. However, PLA’s rigid molecular chains and high crystallinity predispose it to crystallization shrinkage and residual stress accumulation during FDM cooling, manifesting as dimensional deviations, warpage, and interlayer delamination. These defects severely restrict its application in precision components and high-tolerance assemblies, constituting a critical bottleneck for PLA-based FDM toward high-end manufacturing [5,6].

Thermoplastic polyurethane (TPU), a flexible elastomer combining elasticity with processability, exhibits low crystallinity, high toughness, and partial miscibility with PLA, and has been widely adopted for PLA toughening [7,8]. Previous studies demonstrate that PLA/TPU blending effectively modulates crystallization behavior and internal stress distribution, mitigating PLA brittleness while reducing cooling-induced shrinkage, thereby improving dimensional stability [9]. Rahmatabadi et al. [10] systematically investigated the fracture toughness of FDM-printed PLA/TPU blends with different component ratios, reporting that higher PLA content increased ultimate tensile strength and fracture toughness, while higher TPU content led to more printing-induced voids and weaker interlayer bonding. These findings underscore the importance of understanding the compatibility and processing behavior of PLA/TPU blends.

Among diverse toughening approaches for polylactic acid (PLA), thermoplastic polyurethane (TPU) is selected as the blending phase for four key reasons. First, polar interactions between the ester groups of PLA and the urethane segments of TPU deliver intrinsically favorable interfacial compatibility, which strengthens interphase bonding without requiring additional compatibilizers [7,8,11]. Second, TPU shares an identical processing temperature window (190–220 °C) with PLA for standard FDM fabrication, whereas alternative toughening polymers such as polycaprolactone (PCL) and poly(butylene adipate-co-terephthalate) (PBAT) demand drastic adjustments to printing parameters. Third, unlike nanofiller reinforcement systems (nanoclay, carbon nanotubes, cellulose nanofibers, etc.), TPU melt blending avoids filler agglomeration that deteriorates printability. Fourth, the abundant literature on PLA/TPU blends [12,13,14,15] supports reliable cross-comparison between our experimental findings and previous studies. Other candidate toughening polymers are excluded from this work. The primary aim of this study is to construct a systematic multi-objective optimization framework based on a well-characterized blend system, and PLA/TPU remains the most widely researched and industrially promising blend material for FDM manufacturing.

Nevertheless, the significant differences in molecular chain structure and polarity between rigid polyester PLA and flexible polyurethane TPU render blend compatibility highly susceptible to compositional variations. Different blending ratios induce substantial changes in filament microstructure—including phase dispersion homogeneity, interfacial adhesion, crystallinity, and crystalline morphology—which critically govern melt rheological behavior and cooling shrinkage during FDM, ultimately determining the dimensional accuracy of printed parts [11,12,13,14,15].

Despite extensive research on FDM parameter optimization and PLA/TPU blend modification, three critical research gaps remain. First, most studies optimize FDM parameters or PLA/TPU blending ratios separately, overlooking their synergistic effects on dimensional accuracy, which limits the practical utility of optimized results and prevents precise dimensional control [16,17]. Recent studies have investigated interfacial shear strength in multi-material FDM printing [18], yet the coupled effects of blend ratio and printing parameters on overall dimensional accuracy remain insufficiently explored. Second, existing research predominantly focuses on macroscopic dimensional characterization, lacking in-depth correlation analysis between filament microstructure (compatibility, crystallinity) and final part accuracy. The intrinsic mechanism by which blending ratio regulates microstructure and subsequently affects dimensional errors remains insufficiently elucidated [19,20]. For example, the effects of interlayer adhesion [21] and microscale deformation mechanisms [22] on dimensional accuracy have rarely been systematically incorporated into multi-objective optimization frameworks of PLA/TPU blends. Third, most optimization efforts employ single-objective approaches targeting individual dimensional indicators (e.g., length error), failing to simultaneously address multiple dimensional deviations including length, width, inner diameter, and outer diameter, thus precluding comprehensive accuracy control [23].

To address these gaps, multi-criteria decision-making and experimental design methods have emerged as effective pathways for multi-objective optimization of material formulations and process parameters. The entropy weight-TOPSIS method, as a typical objective multi-attribute decision-making approach, can objectively determine indicator weights, effectively avoiding subjective bias and enabling efficient comprehensive performance evaluation, and has been widely applied in FDM process and formulation optimization [24,25]. Orthogonal experimental design efficiently screens significant process parameters affecting dimensional accuracy with reduced experimental runs, substantially lowering workload and cost while improving efficiency [26]. Building upon these methodologies, the present study integrates single-factor experiments, orthogonal array design, and entropy weight–TOPSIS into a synergistic optimization framework.

This study adopts PLA/TPU blended filaments and establishes a multi-objective dimensional accuracy optimization system for FDM, utilizing length, width, inner diameter, and outer diameter deviations as core evaluation indices. A two-step experimental framework is implemented: single-factor experiments combined with the entropy weight–TOPSIS method are first performed to determine the optimal PLA/TPU blend ratio. Based on the optimized composition, an L_16_(4^5^) mixed-level orthogonal experiment is further conducted to systematically investigate the influences of four key printing parameters (printing speed, printing temperature, layer thickness, and infill rate) on dimensional deviation. Range analysis and repeated-measures ANOVA are adopted to quantify parameter significance, while the entropy weight method objectively assigns weights to each dimensional index. Comprehensive TOPSIS scores are finally calculated to screen the optimal parameter combination for high-precision FDM printing.

The scientific novelty of this study lies in three distinct aspects. First, unlike most existing studies that independently optimize either blend composition or processing parameters, this work proposes a hierarchical sequential optimization strategy that first optimizes material formulation and subsequently optimizes printing processes. This material–process collaborative optimization effectively bridges the gap between material modification and precision printing regulation. Second, this study innovatively applies the entropy weight–TOPSIS method to the multi-index dimensional accuracy evaluation of PLA/TPU printed components, providing a fully data-driven and objective solution to balance multiple conflicting dimensional deviation indicators. Third, the systematic integration of range analysis, statistical ANOVA, and experimental validation constructs a robust and reliable optimization framework, which can guide the precision manufacturing of FDM-fabricated polymer blends.

**Figure 1:**
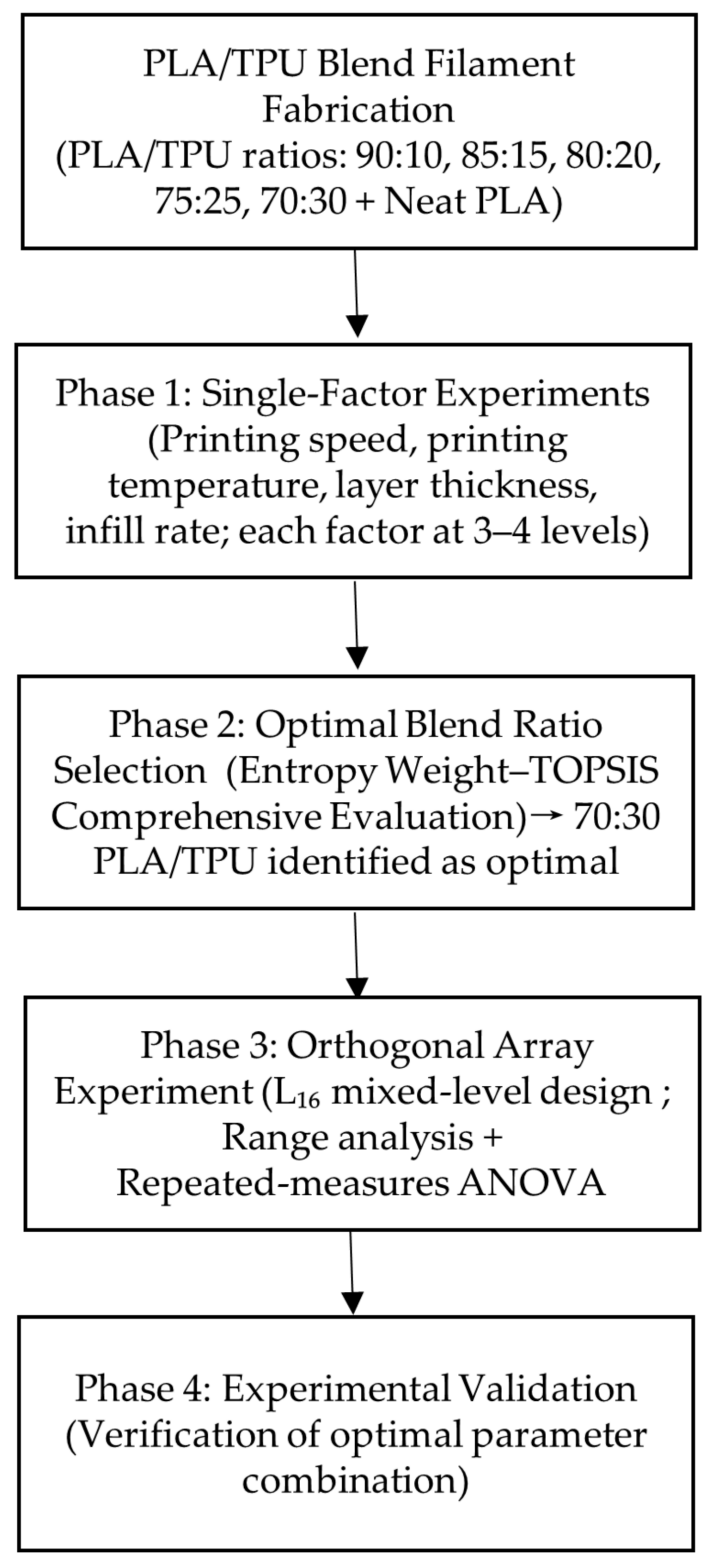
Methodological framework of this study, illustrating the five-stage sequential experimental procedure: (1) material preparation and filament fabrication; (2) single-factor experiments for blend ratio screening; (3) identification of the optimal PLA/TPU blend ratio via entropy weight–TOPSIS; (4) orthogonal array design and ANOVA for process parameter optimization; and (5) experimental validation of the optimized parameters.

The overall experimental workflow of this study is illustrated in Figure 1, which outlines the sequential optimization strategy from material preparation to parameter validation.

The remainder of this paper is structured as follows. Section 2 describes the materials, specimen preparation, experimental design, and characterization methods. Section 3 presents the experimental results, including single-factor experiments, orthogonal array analysis, ANOVA, entropy weight–TOPSIS evaluation, and validation. Section 4 discusses the findings, addresses the underlying mechanisms, acknowledges limitations, and outlines future directions. Section 5 concludes the paper with a summary of key findings and their implications for precision FDM manufacturing of PLA/TPU blends.

## 2. Materials and Methods

### 2.1. Materials

Polylactic acid (PLA), grade 2003D, density 1.4 g/cm^3^, melting point 170 °C, was purchased from NatureWorks LLC (Plymouth, MN, USA). Thermoplastic polyurethane (TPU), grade 95A, density 1.12 g/cm^3^, Shore hardness 90 A, was supplied by BASF SE (Ludwigshafen, Germany).

### 2.2. Equipment and Instruments

The materials preparation and specimen fabrication were carried out using an electronic balance (ML3002, Meilen, Shenzhen, China; 3000 g capacity, 0.01 g readability), an electrical thermostatic drying oven (101-2A, Shaoxing Luyue Instrument Co., Ltd., Shaoxing, China), a micro-conical twin-screw extruder (SJSZ-10A, Wuhan Ruiming Experimental Instrument Co., Ltd., Wuhan, China), and a micro-drawing winder (FQJS-100, Wuhan Ruiming Experimental Instrument Co., Ltd., Wuhan, China). FDM printing was performed using a desktop 3D printer (Bambu Lab P1S, Shenzhen Tuozhu Technology Co., Ltd., Shenzhen, China). Dimensional measurements were conducted using a micrometer screw gauge (701-01, range: 0–25 mm; and 701-02, range: 25–50 mm, Harbin Measuring & Cutting Tool Group Co., Ltd., Harbin, China) and a vernier caliper (range: 0–150 mm, Harbin Measuring & Cutting Tool Group Co., Ltd., Harbin, China). The crystallinity of the specimens was analyzed using an X-ray diffractometer (PANalytical Empyrean, PANalytical, Almelo, The Netherlands). For morphological observations, the cryo-fractured surfaces were sputter-coated with gold using an ion sputtering coater (Hezao GMC-1500, Hezao, Dongguan, China) and subsequently examined using a field-emission scanning electron microscope (Zeiss Sigma300, Zeiss, Oberkochen, Germany).

### 2.3. Sample Preparation

PLA and TPU pellets were weighed using an electronic balance and mechanically blended for 50 min to ensure homogeneous dispersion. The blended mixtures were then dried in an electric thermostatic oven at 60 °C for 8 h to remove residual moisture. Subsequently, the dried PLA/TPU blends with weight ratios of 90:10, 85:15, 80:20, 75:25, and 70:30 were fed into a conical twin-screw extruder for filament fabrication. The extrusion temperatures along the barrel were set to 100, 120, 140, 175, and 180 °C. The melts were extruded and drawn at a screw speed of 20 r/min. The filament diameter was monitored periodically using a micrometer screw gauge, and the winding speed was adjusted manually to maintain the diameter within 1.65–1.75 mm. The fabricated filaments were then wound onto spools using a filament winder.

**Figure 2:**
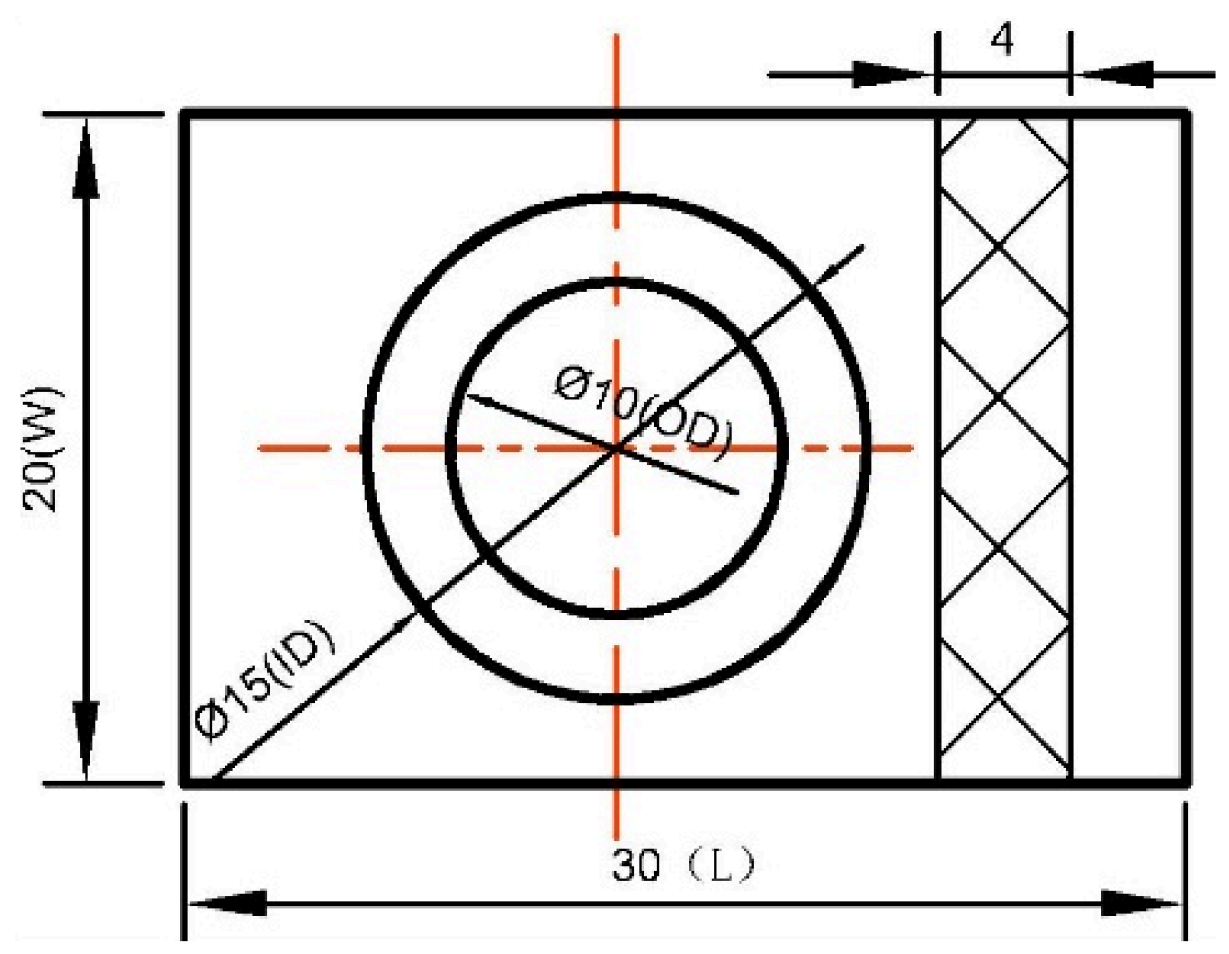
CAD model and nominal dimensions of the specimen (unit: mm). Note: *L*, *W*, *OD*, and *ID* in the figure denote Length, Width, Outer Diameter, and Inner Diameter, respectively, with their corresponding nominal values (in mm) marked in the figure. In the following text, *L*, *W*, *OD*, and *ID* refer to the measured deviations of the above dimensions.

**Figure 3:**
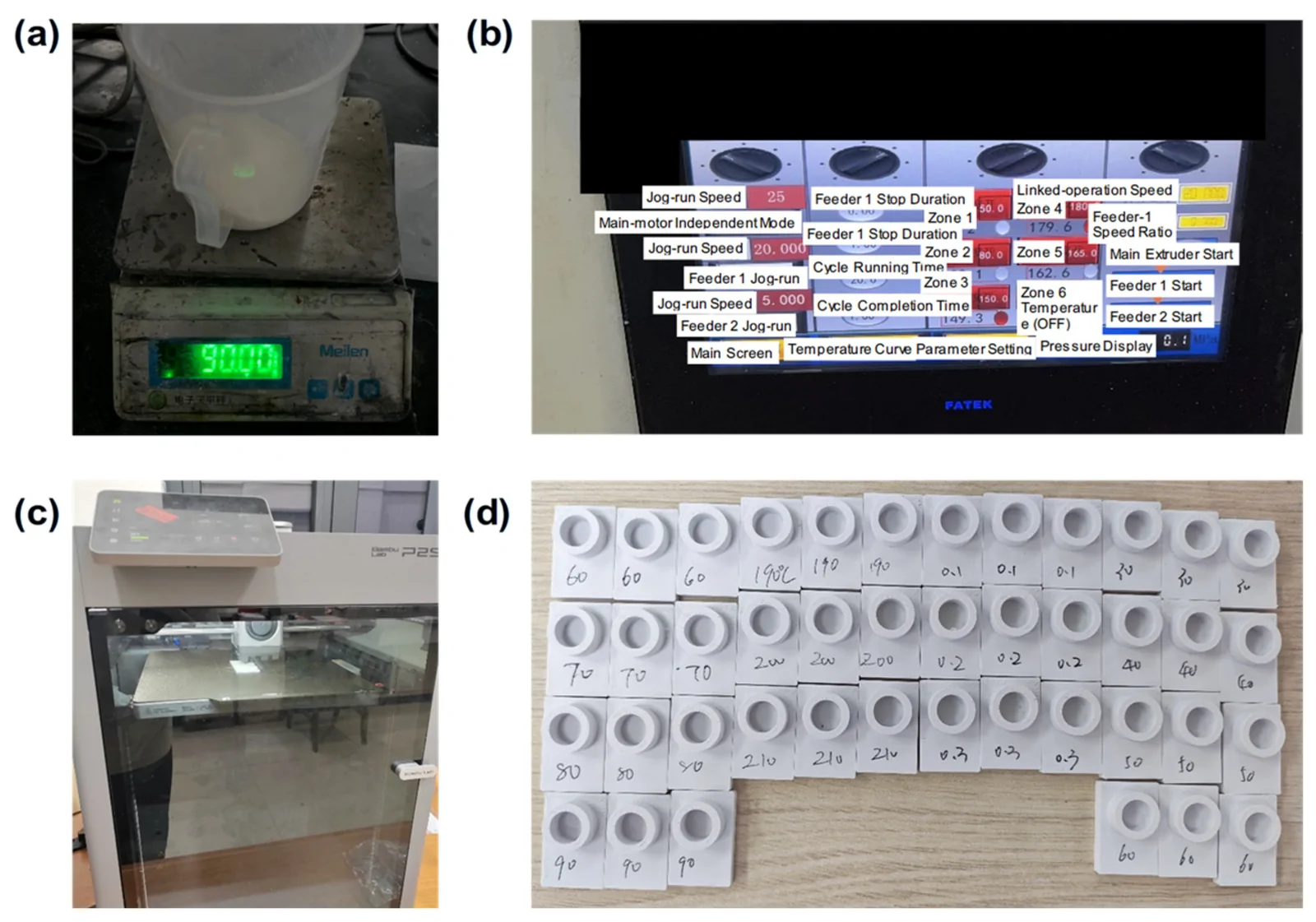
Fabrication process of specimens: (**a**) raw material weighing; (**b**) filament extrusion via twin-screw extruder; (**c**) FDM printing process; (**d**) printed specimens (three replicates per group, arranged by different processing levels).

FDM specimens were then printed using the as-prepared filaments (Figure 2) [27]. After printing completion, the specimens were naturally cooled within the enclosed chamber for approximately 40 min before being removed. All specimens were conditioned at ambient temperature for 24 h prior to dimensional measurements to ensure complete relaxation and eliminate residual thermal effects. Three replicates were printed for each experimental condition. The overall experimental procedure is illustrated in Figure 3.

### 2.4. Performance Testing and Structural Characterization

#### 2.4.1. Dimensional Measurement

The length, width, and outer diameter of the specimens were measured using a micrometer screw gauge (accuracy: 0.01 mm), while the inner diameter was measured using a vernier caliper (accuracy: 0.02 mm). Five specimens from each group were measured, and the results were averaged.

#### 2.4.2. X-Ray Diffraction (XRD) Analysis

X-ray diffraction (XRD) analysis was performed on the specimens using an X-ray diffractometer to investigate the crystalline structure. The measurements were conducted under the following conditions: Cu Kα radiation (λ = 0.15418 nm), generator voltage of 40 kV, current of 40 mA, scanning range of 2θ = 5–90°, scanning speed of 10°/min, and cumulative scanning count of 16 times. The crystallinity ($X_{c}$) of the specimens was calculated using Equation (1),

(1) $$X_{c}=\frac{A_{c}}{A_{c}+KA_{a}}\times 100\%$$

where $A_{c}$ and $A_{a}$ are the integrated intensities of the crystalline diffraction peaks and the amorphous halo, respectively, over the integration range, and *K* is the experimental calibration factor.

#### 2.4.3. Scanning Electron Microscopy (SEM) Observation

The morphology of the cryo-fractured surfaces of the specimens was observed using a scanning electron microscope. Prior to observation, the cryo-fractured samples were mounted onto conductive sample holders and sputter-coated with gold using an ion sputter coater (sputtering time: 30 s, current: 10 mA) to eliminate charge accumulation on the surface of the non-conductive materials. The micrographs were acquired using a secondary electron detector at an acceleration voltage of 5 kV.

### 2.5. Experimental Design and Procedure

A combined approach of single-factor experiments and orthogonal array testing was adopted to systematically investigate the effects of key process parameters and PLA/TPU blend ratios on the dimensional accuracy of FDM-printed parts. Dimensional deviation served as the primary response variable, and the entropy weight–TOPSIS method was integrated to enable synergistic optimization of material formulation, printing parameters, and dimensional precision.

Phase I: Single-factor pre-experiments. Following the principle of controlled variables, this phase examined the individual effects of printing temperature, printing speed, layer height, filling rate, and PLA/TPU blend ratio on the dimensional deviation of printed specimens. For each blend ratio, the entropy weight–TOPSIS method was first applied to determine the optimal combination of process parameters through multi-objective optimization. Subsequently, the same method was used to perform a comprehensive evaluation across all ratios to identify the globally optimal PLA/TPU blend ratio.

Phase II: Orthogonal optimization experiments. Since single-factor experiments do not account for parameter interactions or the coupled effects between blend ratio and printing conditions, orthogonal array experiments were conducted based on the globally optimal blend ratio. Range analysis and analysis of variance (ANOVA) were performed to systematically evaluate the significance of each process parameter. This allowed further refinement of the printing parameters under the optimal blend ratio and validation of the reliability of the optimization model.

While orthogonal array testing primarily captures the main effects of individual factors without estimating interactions between parameters (e.g., printing temperature × printing speed), this design was intentionally selected for its screening efficiency. An L_16_ mixed-level orthogonal array was adopted—with factors *A* and *C* at 4 native levels, and factors *B* and *D* extended from 3 to 4 levels via the pseudo-level method—requiring only 16 runs, versus 256 runs for a full factorial design, while the unassigned fifth column provides degrees of freedom for error estimation. Given the experimental scope—five blend ratios and four printing parameters—this approach offers a practical balance between feasibility and screening effectiveness. Our ongoing work will employ response surface methodology to systematically investigate interaction effects and further refine the optimization.

## 3. Results

### 3.1. Design and Implementation of Single-Factor Experiments

#### 3.1.1. Design of the Single-Factor Experimental Scheme

To investigate the effects of the PLA/TPU blend ratio and key process parameters on the dimensional deviations of FDM-printed parts, single-factor experiments were performed. The factors examined included the PLA/TPU blend ratio (mass ratios of 90:10, 85:15, 80:20, 75:25, and 70:30), printing speed (*A*), printing temperature (*B*), infill rate (*C*), and layer height (*D*). The dimensional deviations in length (*L*), width (*W*), inner diameter (*ID*), and outer diameter (*OD*) of the printed specimens served as the response variables. The control variable method was adopted, i.e., only one factor was varied at a time while the other parameters and the blend ratio were kept constant, allowing the individual effect of each factor on dimensional deviation to be isolated. By systematically analyzing the response patterns of *L*, *W*, *ID*, and *OD* under varying factor levels, the significance and trends of the influence of each process parameter on the dimensional accuracy of FDM-printed parts were determined. The factor levels for each parameter are listed in Table 1, and all specimens were printed accordingly.

#### 3.1.2. Single-Factor Experimental Results and Analysis

**Figure 4:**
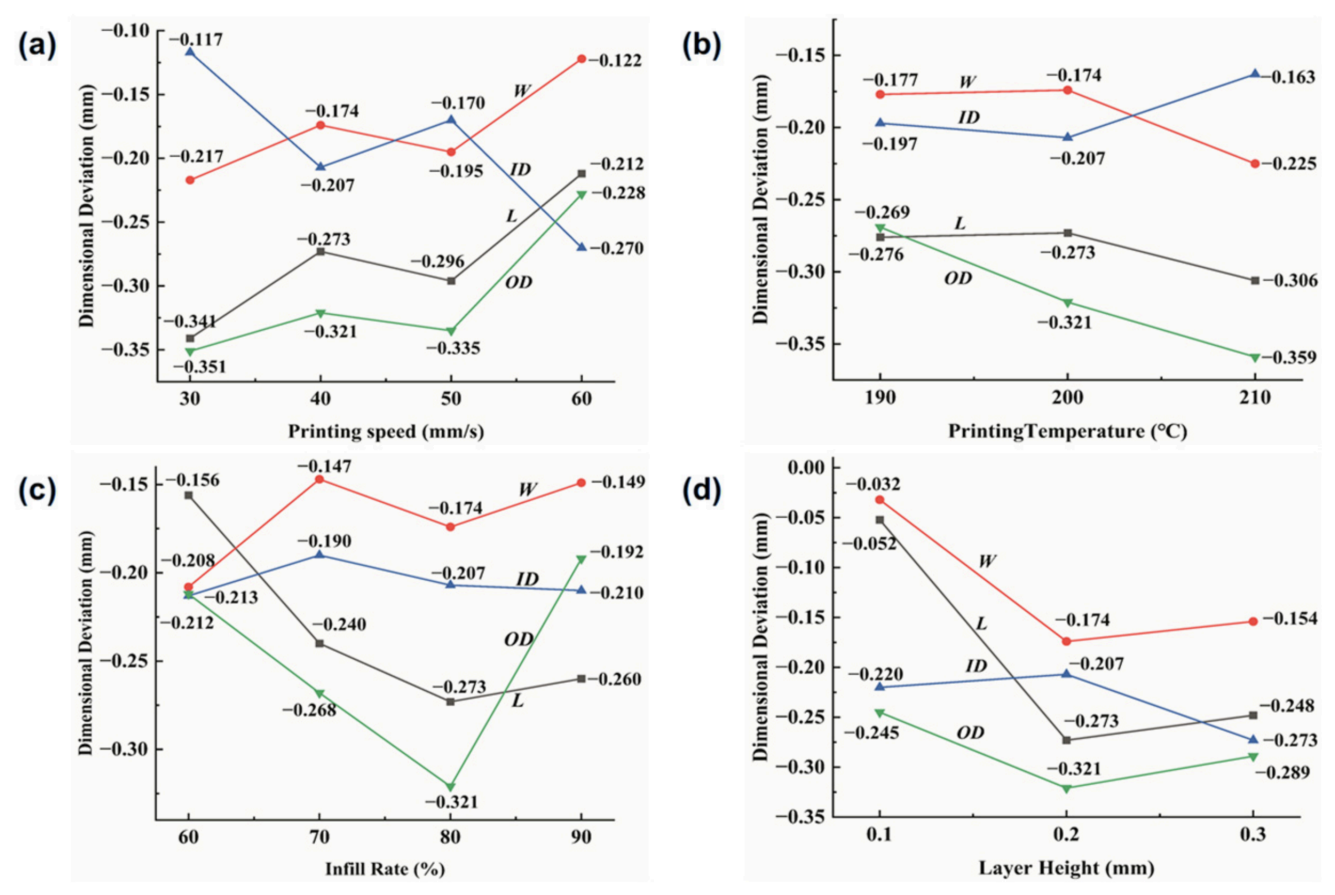
Effects of process parameters on dimensional deviations of PLA/TPU (70:30) blend under single-factor experiments: (**a**) printing speed; (**b**) printing temperature; (**c**) infill rate; (**d**) layer thickness. The dimensional deviations of *L* (length), *W* (width), *ID* (inner diameter), and *OD* (outer diameter) are represented by black, red, blue, and green lines, respectively. Each subplot adopts an independent vertical axis range matching its own data distribution to clearly display the variation trend of each dimensional deviation. Data are presented as the mean (*n* = 3).

**Figure 5:**
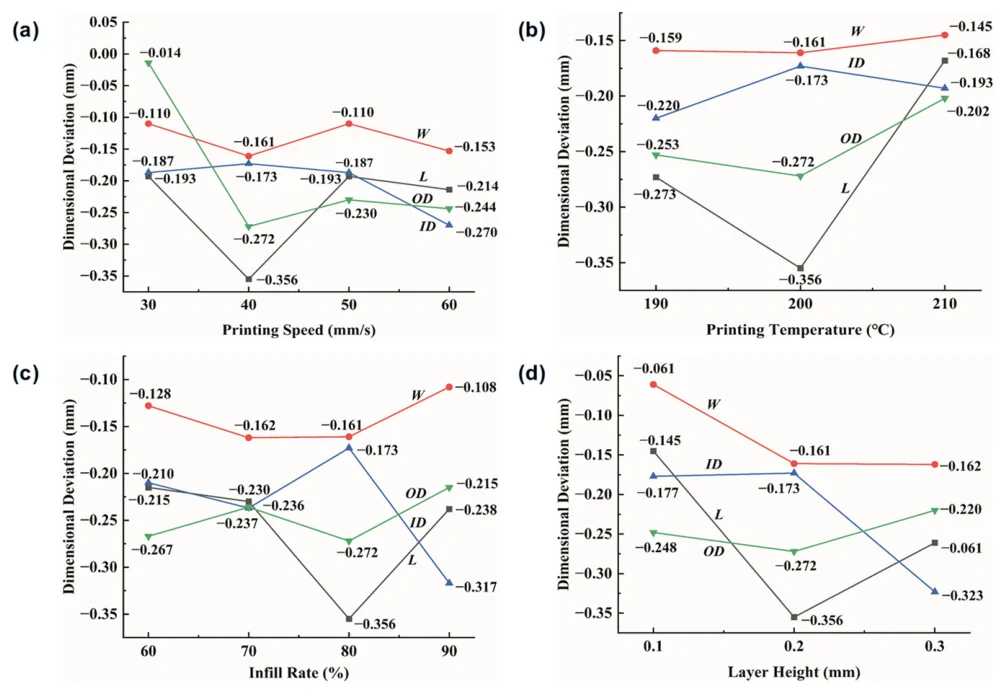
Effects of different process parameters on dimensional deviations of printed specimens with a PLA/TPU blend ratio of 75:25: (**a**) printing speed; (**b**) printing temperature; (**c**) infill rate; (**d**) layer height. Legends and axis settings follow those in Figure 4.

**Figure 6:**
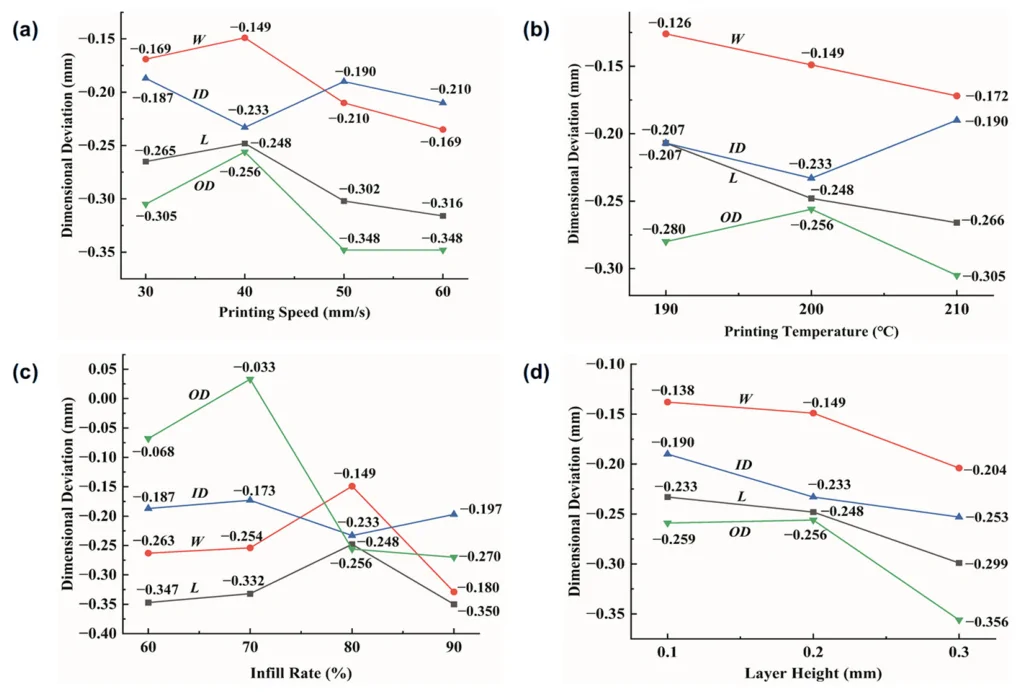
Effects of different process parameters on dimensional deviations of printed specimens with a PLA/TPU blend ratio of 80:20: (**a**) printing speed; (**b**) printing temperature; (**c**) infill rate; (**d**) layer height. Legends and axis settings follow those in Figure 4.

**Figure 7:**
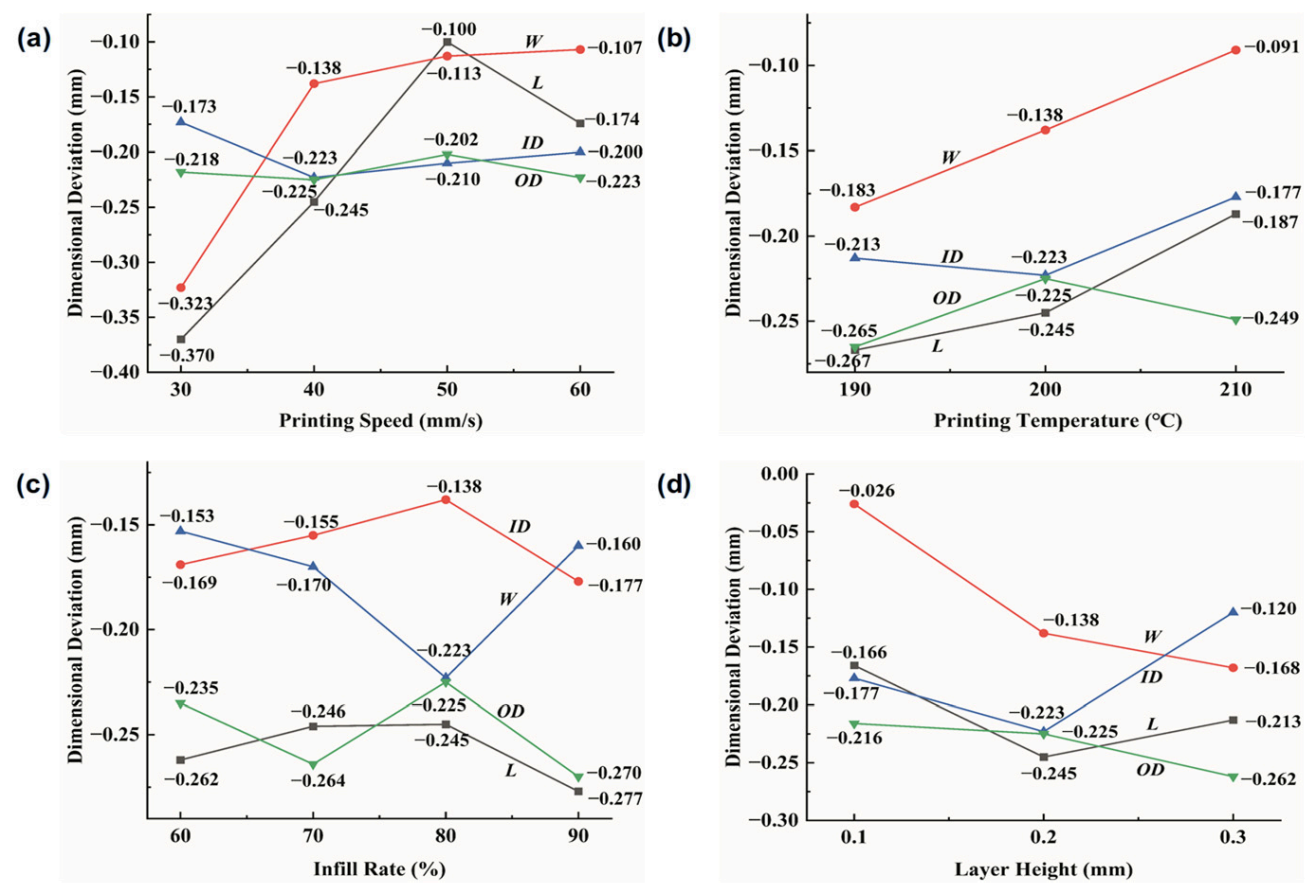
Effects of different process parameters on dimensional deviations of printed specimens with a PLA/TPU blend ratio of 85:15: (**a**) printing speed; (**b**) printing temperature; (**c**) infill rate; (**d**) layer height. Legends and axis settings follow those in Figure 4.

**Figure 8:**
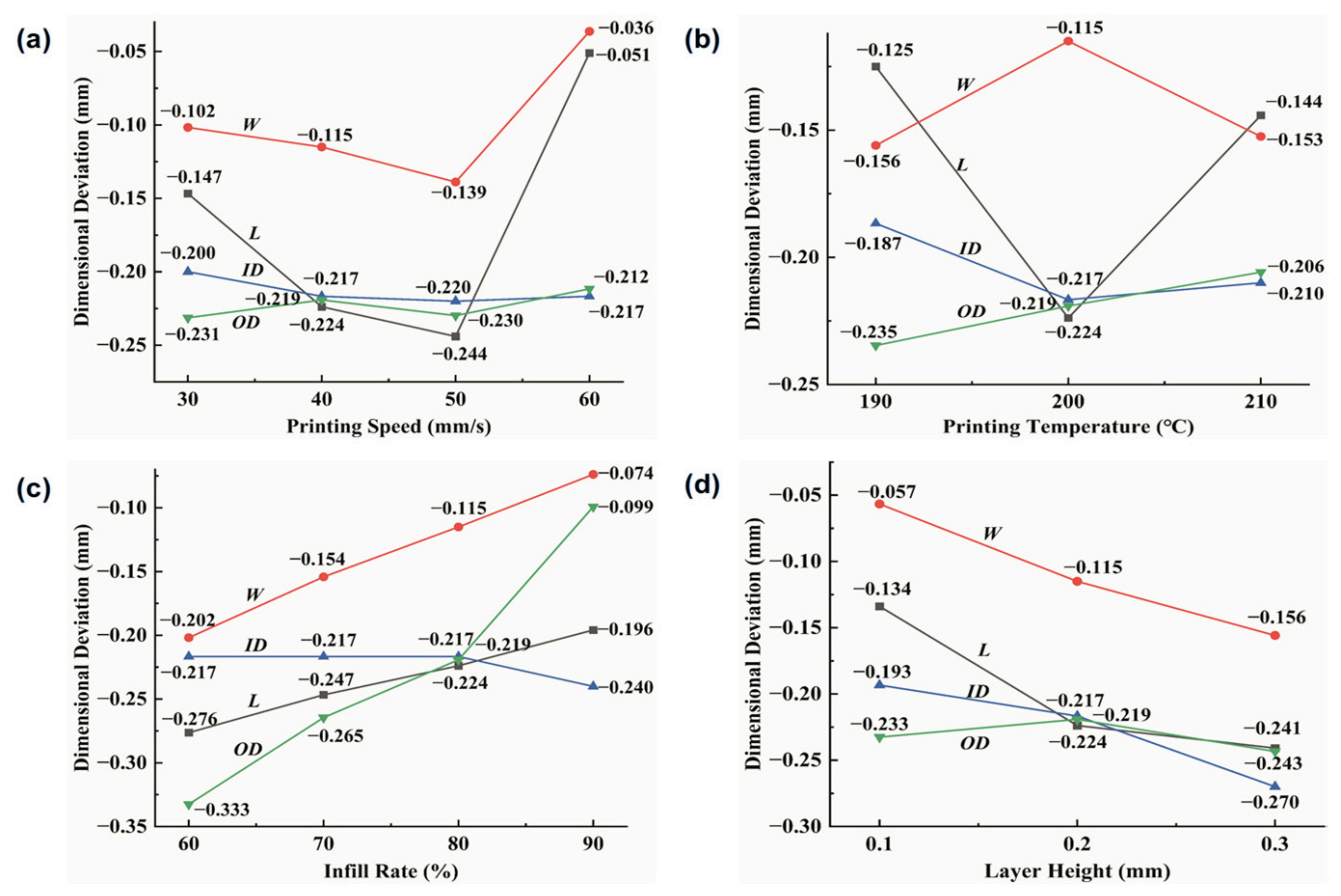
Effects of different process parameters on dimensional deviations of printed specimens with a PLA/TPU blend ratio of 90:10: (**a**) printing speed; (**b**) printing temperature; (**c**) infill rate; (**d**) layer height. Legends and axis settings follow those in Figure 4.

**Figure 9:**
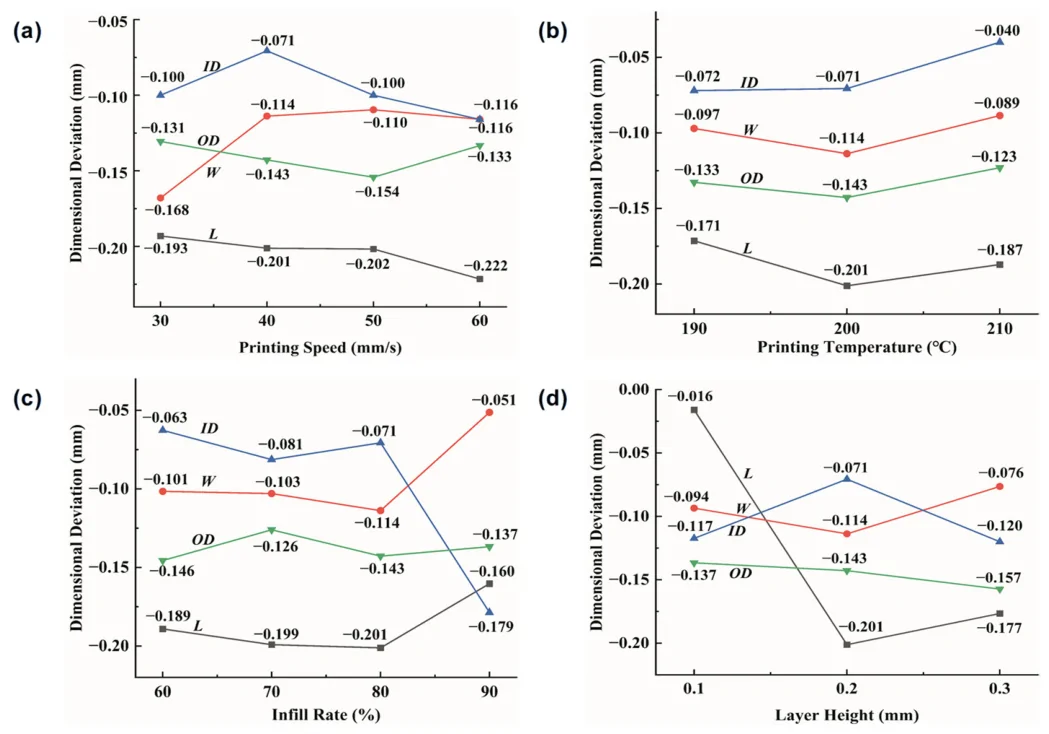
Effects of different process parameters on dimensional deviations of neat PLA: (**a**) printing speed; (**b**) printing temperature; (**c**) infill rate; (**d**) layer height. Legends and axis settings follow those in Figure 4.

Figure 4, Figure 5, Figure 6, Figure 7, Figure 8 and Figure 9 illustrate the effects of printing speed, printing temperature, infill rate, and layer height on the dimensional deviations of FDM-printed specimens across six material systems: five PLA/TPU blend ratios (70:30, 75:25, 80:20, 85:15, and 90:10) and neat PLA. All measured dimensional deviations—length (*L*), width (*W*), inner diameter (*ID*), and outer diameter (*OD*)—were negative, indicating shrinkage during cooling and solidification, with smaller absolute values corresponding to higher dimensional accuracy.

Figure 4 presents the dimensional deviation patterns for the PLA/TPU = 70:30 blend ratio. Among the four measured dimensions, *W* exhibited the highest accuracy, with deviations ranging from −0.225 to −0.032 mm across all process parameters, demonstrating the smallest fluctuation amplitude and the most stable response. In contrast, *OD* showed the poorest accuracy, with deviations ranging from −0.359 to −0.192 mm, reflecting significantly greater shrinkage than the other dimensions. *L* and *ID* showed intermediate accuracy between these two extremes.

The superior accuracy of *W* can be attributed to its alignment with the continuous, straight melt deposition path, which ensures uniform stress distribution and mitigates anisotropic shrinkage. In contrast, *OD*, formed by segmented linear toolpaths approximating a circular contour, is subject to cumulative trajectory errors. Additionally, its direct exposure to ambient convection leads to faster edge cooling and non-uniform temperature fields, inducing differential shrinkage and edge warpage, and ultimately greater dimensional deviations [28,29].

As printing temperature, infill rate, and layer height increased, thermal shrinkage intensified across all dimensions, with *OD* being the most sensitive to process parameter variations and *W* being the least sensitive. This hierarchy is consistent with the anisotropic shrinkage mechanism commonly reported in FDM-fabricated parts [30].

Figure 5 presents the dimensional deviations for the PLA/TPU = 75:25 blend ratio. Compared with the 70:30 ratio, overall shrinkage and fluctuation ranges were reduced, while the effects of process parameters followed similar trends, albeit with notably mitigated thermal shrinkage and improved dimensional stability.

Three key differences were identified. (1) *W* deviations were further reduced to a stable range of −0.162 to −0.061 mm, indicating better melt uniformity and more effective suppression of anisotropic shrinkage. (2) *L* showed significantly increased sensitivity to process parameters, with a sharp deviation of −0.356 mm at 40 mm/s, 200 °C, and 0.2 mm, suggesting that the blend ratio amplified the effects of path errors and interlayer stress on linear dimensions. (3) *OD* deviations decreased slightly overall (ranging from −0.272 to −0.014 mm) but exhibited more drastic responses to infill rate and layer height, both reaching −0.272 mm at 80% infill rate and 0.2 mm layer height, respectively, indicating a narrowed processing window for contour dimensions.

Figure 6 presents the effects of process parameters on dimensional deviations for the PLA/TPU = 80:20 blend ratio. Compared with the 75:25 ratio, overall shrinkage was further reduced, accompanied by improved dimensional stability.

At 40 mm/s, *L*, *W*, and *OD* all reached their minimum values simultaneously (−0.248 mm, −0.149 mm, and −0.256 mm, respectively), indicating this speed as the optimal setting for overall accuracy. At 200 °C, *OD* deviation was minimized, while *L* and *W* showed slight increases but remained within a controllable range. Infill rate exhibited a trade-off: 70% effectively suppressed *OD* shrinkage, whereas 80% yielded optimal linear dimensional deviations; 90% led to a notable increase in all deviations. Regarding layer height, 0.2 mm resulted in the lowest *OD* deviation—achieving a balance between efficiency and precision—while 0.3 mm markedly exacerbated anisotropic shrinkage distortion.

Overall, the 80:20 blend ratio exhibited improved melt extrusion uniformity and more effective anisotropic shrinkage suppression, resulting in enhanced stability and a wider processing window compared with the 75:25 ratio.

Figure 7 presents the effects of process parameters on dimensional deviations for the PLA/TPU = 85:15 blend ratio. As TPU content decreased, the material exhibited enhanced rigidity and significantly reduced thermal shrinkage, leading to improved dimensional accuracy compared with blends containing higher TPU fractions.

As printing speed increased from 30 to 60 mm/s, L and W deviations decreased gradually, while *ID* and *OD* remained relatively stable. However, in the low-speed range (≤40 mm/s), *L* fluctuated considerably, with a range of 0.125 mm between 30 and 40 mm/s—the most severe low-speed instability observed across all blend ratios—whereas *OD* showed almost no variation under the same conditions. At 200 °C, all deviations remained low (*W*: −0.138 mm). Notably, increasing temperature to 210 °C mitigated *W* shrinkage distortion, reducing its deviation from −0.183 to −0.091 mm, a counterintuitive trend suggesting that moderate heating alleviates width-direction non-uniform cooling. As infill rate increased from 60% to 80%, linear deviations were progressively optimized, with L reaching its minimum (−0.245 mm) at 80%, beyond which deviations rebounded. Increasing layer height from 0.1 to 0.3 mm gradually intensified interlayer shrinkage, with absolute *OD* deviation increasing by approximately 21.3%; nonetheless, the overall error level remained lower than that of specimens with higher TPU content.

Overall, anisotropic shrinkage suppression improved with decreasing TPU fraction, and both dimensional control and processing adaptability were superior to those of blends with higher TPU content.

Figure 8 presents the effects of process parameters on dimensional deviations for the PLA/TPU = 90:10 blend ratio. This blend exhibited the highest rigidity and the weakest thermal shrinkage among all formulations, resulting in the best overall dimensional stability.

The optimal conditions for each parameter were as follows. A layer height of 0.1 mm yielded the minimum *W* deviation (−0.057 mm), while 0.2 mm resulted in relatively low fluctuations in *L* and circular feature dimensions (*ID* and *OD*). At 60 mm/s, *W* deviation was minimized (−0.036 mm), whereas 40 mm/s produced the smallest *ID* shrinkage. At 200 °C, all deviations remained most stable across all dimensions, with only a slight increase in lateral deviations upon further temperature elevation. An infill rate of 70% minimized *OD* shrinkage, and notably, this was achieved without requiring stringent internal stress control—unlike other blend ratios where strict parameter constraints were necessary. This “robustness” is a key advantage of the 90:10 formulation.

Overall, this blend ratio consistently exhibited the lowest dimensional deviations across all parameter combinations among the five formulations tested, demonstrating the best single-factor forming accuracy and the widest processing window.

Figure 9 presents the effects of process parameters on dimensional deviations of neat PLA specimens. Owing to its higher rigidity and more pronounced thermal shrinkage compared with the PLA/TPU blends, neat PLA exhibited greater overall deviations than all blend formulations—establishing a baseline that demonstrates the necessity of TPU incorporation for shrinkage mitigation.

Two distinctive features were observed. First, layer height sensitivity was extreme: 0.1 mm yielded the optimal *L* deviation (−0.016 mm), but increasing to just 0.2 mm caused a sharp jump to −0.201 mm—a 12-fold increase. *ID* and *OD*, however, remained largely insensitive to layer height changes. Second, speed sensitivity followed contrasting trends: 40 mm/s minimized *ID* shrinkage (−0.071 mm), yet *L* deviation increased continuously with rising speed, indicating direction-dependent sensitivity. At 200 °C, overall deviations were most stable, with only slight increases at higher temperatures. At an infill rate of 60%, *ID* deviation reached its minimum (−0.063 mm), beyond which it followed a non-monotonic pattern (first increasing then decreasing), while L remained consistently high at low infill rates.

Overall, neat PLA dimensional deviations were most sensitive to layer height and printing speed, with *ID* and *OD* showing better stability than *L* and *W*—the opposite hierarchy observed in blend formulations.

### 3.2. Synergistic Optimization of Process Parameters and Blend Ratio Based on the Entropy Weight–TOPSIS Method

#### 3.2.1. Framework and Methodology of Synergistic Optimization

Among the five blend ratios, PLA/TPU = 90:10 exhibited the lowest shrinkage in length and inner diameter deviations, yet showed considerable deviation in width, indicating a trade-off among dimensional quality indicators. The 70:30 blend, by contrast, exhibited relatively large deviations in length and inner diameter, but performed competitively in width—a pattern that would later favor its overall balance in the TOPSIS evaluation. The 85:15, 80:20, and 75:25 ratios demonstrated intermediate performance with varying trade-off characteristics. Neat PLA, owing to its significant thermal shrinkage, exhibited higher overall dimensional deviations than all blend formulations.

Given that single-factor experiments can only reflect the individual effects of process parameters on dimensional accuracy—without capturing multi-parameter synergies or enabling comprehensive evaluation across different material systems—the entropy weight–TOPSIS method was introduced as a multi-objective optimization approach. Unlike simple weighted summation, TOPSIS evaluates each alternative by its geometric distance to both the positive-ideal and negative-ideal solutions, thereby identifying the option with the most balanced performance across all criteria. This feature is particularly suited to addressing trade-offs among multiple dimensional accuracy requirements: a formulation that excels in one metric but underperforms in another will not necessarily achieve the highest overall ranking. Through objective weighting and comprehensive evaluation, the method enables precise matching between process parameters and material formulations, offering a robust decision-support tool for quality control in FDM manufacturing.

#### 3.2.2. Screening of Optimal Process Parameters for Individual Material Systems

For the six material systems—comprising five PLA/TPU blend ratios and neat PLA—a unified entropy weight–TOPSIS optimization model was employed to identify the optimal printing process parameters for each system, ensuring the best forming quality across all material formulations.

The PLA/TPU = 90:10 blend ratio was selected as a representative case to illustrate the parameter optimization procedure in detail. Following the principle of factor-independent weighting, the entropy weights of four key process parameters—printing speed, printing temperature, infill rate, and layer height—were calculated separately. These were then integrated with the TOPSIS method to perform a comprehensive evaluation of all process parameter combinations [17,18]. The optimal parameter combination for this blend ratio was determined as: printing speed of 50 mm/s, printing temperature of 200 °C, infill rate of 60%, and layer height of 0.3 mm. Under this combination, the printed specimens achieved the highest dimensional accuracy and the mildest shrinkage behavior, which can serve as the standard process scheme for this formulation.

The same optimization logic was applied to the remaining five material systems (70:30, 75:25, 80:20, 85:15, and neat PLA). For each system, the entropy weight method was used to determine the weights of the process parameters, followed by comprehensive evaluation via TOPSIS to identify the corresponding optimal parameter combination. The optimal process parameters for all material systems are summarized in Table 2.

As shown in Table 2, the ophe optimal process parameter combinations varied considerably across the six material systems (five PLA/TPU blend ratios and neat PLA), indicating that the material formulation exerts a significant influence on the selection of optimal printing parameters. Therefore, to achieve the best forming quality for each material system, the four key process parameters—printing speed, printing temperature, infill rate, and layer height—should be adjusted accordingly.

For each material system, the optimal dimensional deviations corresponding to the identified parameter combinations all fell within the ranges observed in the respective single-factor experiments. Furthermore, the coefficients of variation (*CV*) across three repeated measurements were all below 5%, satisfying the requirement for experimental reliability. These results confirm that the selected optimal process parameters for each material system are reasonable and stable, and can therefore be used for the subsequent determination of the globally optimal formulation.

#### 3.2.3. Determination of the Globally Optimal Material System

Based on the measured dimensional deviation data for the six material systems (five PLA/TPU blend ratios and neat PLA) obtained under their respective optimal process parameters, the entropy weight–TOPSIS coupled model was employed to conduct a quantitative comprehensive evaluation of the forming quality for each material system, aiming to identify the globally optimal formulation. The entropy weight method was used to determine the objective weights of the four evaluation indicators—*L*, *W*, *ID*, and *OD*—which were then integrated with the TOPSIS model to calculate the comprehensive evaluation score for each material system. These scores were subsequently ranked, and the system with the highest score was identified as the globally optimal material system. The corresponding evaluation results are presented in Table 3.

Table 3 shows that the four indicator weights are all close to 0.25, indicating no significant difference in their individual influence on forming quality. The 70:30 blend achieves the highest $C_{i}$ of 0.680—a result explained by its balanced dimensional response. Although the 90:10 blend outperforms others in length and inner diameter deviations, its width deviation (−0.201 mm) ranks among the poorest, revealing a critical trade-off between dimensional quality indicators. In contrast, the 70:30 blend, while never ranking first in any single metric, maintains moderate-to-high performance across all four dimensions without any significant weakness, with $D_{i}^{+}$ = 0.202 and $D_{i}^{-}$ = 0.428 reflecting its favorable TOPSIS geometry. Under the TOPSIS framework, which favors balanced performance over isolated excellence, this uniform profile yields the highest overall ranking. To validate the robustness of this ranking, a sensitivity analysis was performed using equal weighting (all four indicators weighted equally). The Spearman rank correlation coefficient between the entropy-based and equal-weight rankings was 1.0, and the 70:30 blend remained the top-ranked formulation under both schemes, confirming that the optimality of the 70:30 blend is independent of the specific weighting approach. The 70:30 PLA/TPU blend is therefore identified as the globally optimal formulation.

#### 3.2.4. X-Ray Diffraction Analysis

**Figure 10:**
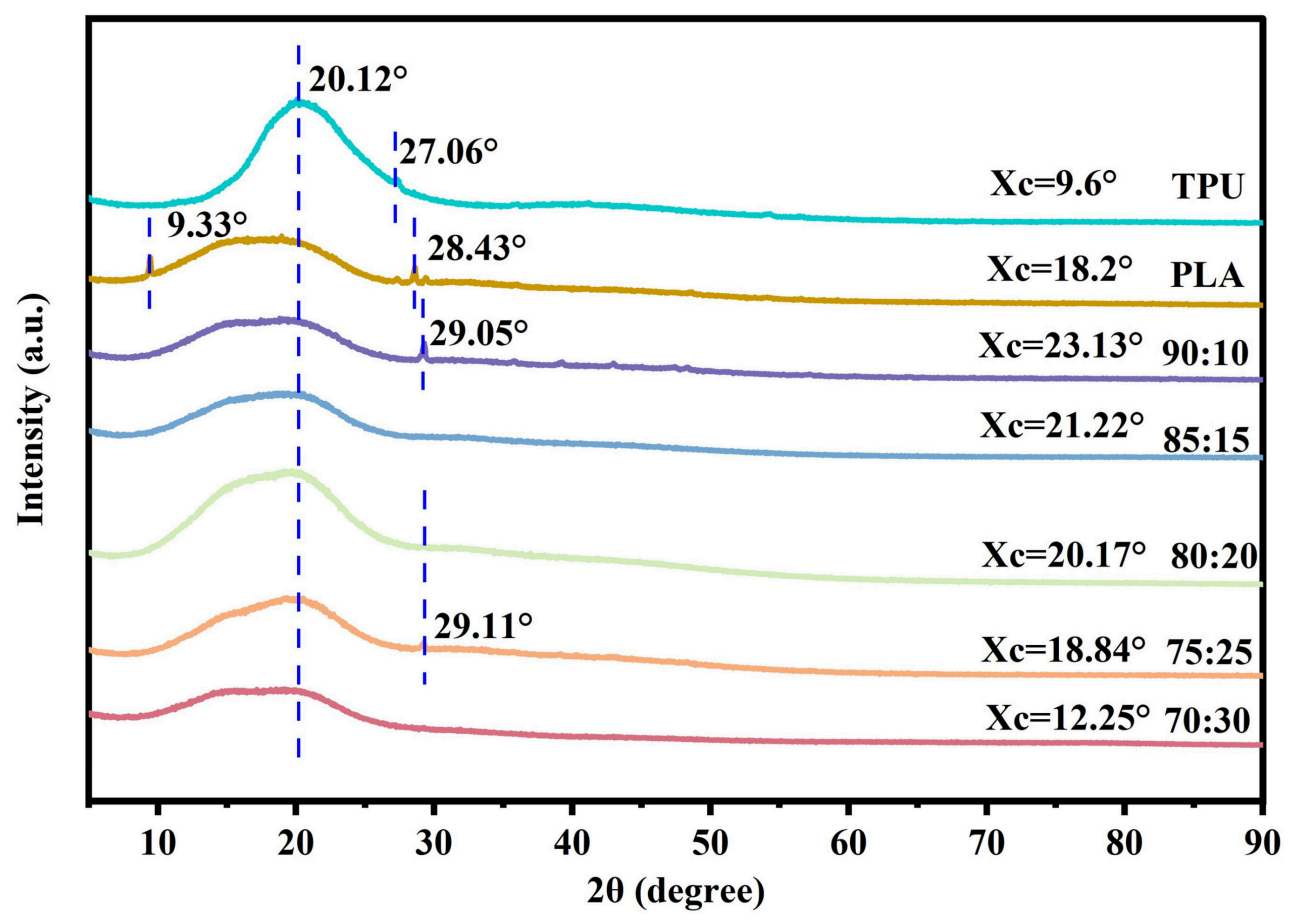
XRD patterns of neat PLA, neat TPU, and PLA/TPU blend filaments at different weight ratios (90:10, 85:15, 80:20, 75:25, and 70:30). The inset table summarizes the crystallinity values derived from the integrated peak areas.

The XRD patterns of neat PLA, neat TPU, and their blends are shown in Figure 10. Neat PLA exhibits a distinct crystalline diffraction peak at 2*θ* = 28.43°, accompanied by a minor peak at 2*θ* = 9.33°, characteristic of the typical α-crystalline structure of PLA [31]. Neat TPU displays a broad diffuse diffraction halo centered at 2*θ* = 20.12° with a weak shoulder around 27.06°, which arises from the ordered packing of the hard-segment domains within the soft-segment matrix via microphase separation—a feature typical of weakly crystalline elastomers, consistent with its measured crystallinity of 9.6% [32].

For all PLA/TPU blends, no new diffraction peaks were observed, indicating that blending occurs solely at a physical level without chemical reaction between the two phases. This suggests that PLA and TPU remain immiscible or partially miscible, with no co-crystallization taking place. Notably, the principal diffraction peak of the PLA phase shifts slightly toward higher 2*θ* values with increasing TPU content, implying a reduction in the interchain spacing of PLA—likely due to the intercalation of TPU molecular chains into the PLA matrix, which disrupts the original chain packing.

The crystallinity of the blends exhibits a non-monotonic dependence on TPU content. At a low TPU fraction (90:10), the crystallinity increases from 18.2% (neat PLA) to 23.1%, suggesting that the dispersed TPU domains act as heterogeneous nucleation sites that promote PLA crystallization [33]. However, as the TPU proportion increases further (85:15, 80:20, 75:25), the crystallinity gradually decreases to 21.2%, 20.2%, and 18.8%, respectively. This decline is attributed to the spatial hindrance imposed by interpenetrating TPU molecular chains, which impede the segmental mobility of PLA and restrict crystal growth. At the highest TPU content examined (70:30), the crystallinity drops sharply to 12.3%, coinciding with a phase inversion in which TPU becomes the continuous matrix. This substantial reduction further confirms that an excessive TPU fraction suppresses PLA crystallization [32].

#### 3.2.5. Microstructural Mechanism Corroboration Analysis

**Figure 11:**
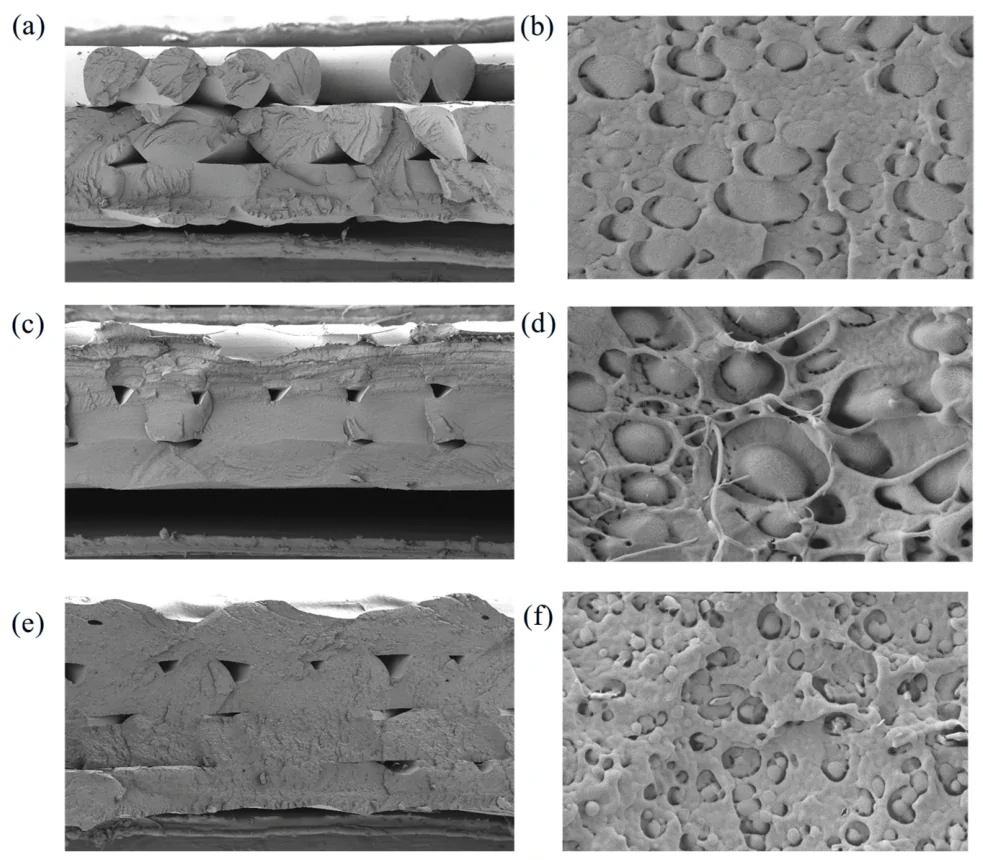
SEM micrographs of 3D-printed neat PLA and 7:3 PLA/TPU blend specimens: (**a**,**b**) neat PLA fabricated with the optimal PLA printing parameters; (**c**,**d**) 7:3 PLA/TPU blend fabricated with the optimal blend printing parameters; (**e**,**f**) 7:3 PLA/TPU blend fabricated with the optimal PLA printing parameters. Scale bars: (**a**,**c**,**e**) = 200 μm; (**b**,**d**,**f**) = 500 nm.

Compared with the neat PLA specimens (Figure 11a,b), which exhibit a smooth and featureless fracture surface characteristic of brittle fracture, the 7:3 PLA/TPU blend prepared under blend-optimized conditions (Figure 11c,d) displays a distinct two-phase “sea-island” morphology, where discrete TPU domains are uniformly dispersed within the continuous PLA matrix. This morphological transition confirms that the incorporation of TPU effectively disrupts the homogeneous structure of neat PLA, leading to a typical immiscible or partially miscible polymer blend morphology [34].

In contrast, the blend fabricated with PLA-optimized parameters (Figure 11e,f) exhibits considerably larger and more aggregated TPU particles, along with pronounced interfacial debonding and visible voids resulting from particle pull-out, indicating poor interfacial adhesion between the two phases [35]. By comparison, the blend-optimized sample (Figure 11c,d) shows substantially finer TPU domains with a more homogeneous dispersion and significantly reduced interfacial gaps. Moreover, localized fibrillar structures or micro-cracks at the phase boundaries suggest enhanced interfacial compatibility and mechanical interlocking.

Collectively, these microstructural observations demonstrate that optimizing printing parameters specifically for the blend system is essential for refining the TPU dispersed phase and achieving superior interfacial adhesion. The finer and more uniform dispersion of TPU domains, together with the elimination of interfacial defects, promotes more homogeneous melt flow behavior during extrusion, facilitates consistent layer-by-layer deposition, and reduces internal voids and non-uniform solidification shrinkage—all of which are critical factors directly governing the dimensional accuracy of the printed parts. In contrast, the coarse and aggregated TPU domains and poor interfacial bonding observed under PLA-optimized conditions (Figure 11e,f) are likely to cause non-uniform melt rheology and localized stress concentrations during solidification, thereby exacerbating dimensional deviations and compromising geometric fidelity. Therefore, tailoring printing parameters to the specific blend composition is not only fundamental to morphological refinement but also a prerequisite for achieving high-precision 3D-printed PLA/TPU blend components [36]. This morphological interpretation is fully consistent with the crystallinity trends revealed by XRD analysis, where the 70:30 blend exhibited a substantially reduced crystallinity (12.3%) due to phase inversion, further confirming that the composition-dependent phase structure and process-induced morphology collectively govern the dimensional accuracy of the printed parts.

#### 3.2.6. Summary of Microstructural Mechanisms

The TOPSIS evaluation, XRD analysis, and SEM observations collectively confirm that the 70:30 blend ratio exhibits the best overall dimensional accuracy. The TOPSIS results show that the 70:30 ratio achieves the highest relative closeness of 0.680, indicating the most balanced performance across the four measured dimensions. XRD reveals that this ratio possesses the lowest crystallinity (12.3%) among all blends, implying minimal and more uniform solidification shrinkage during printing. SEM further demonstrates that the 70:30 printed parts, processed under blend-matched parameters, exhibit the finest TPU dispersion, strongest interfacial adhesion, and fewest interfacial defects, ensuring homogeneous stress distribution during cooling and eliminating localized strain concentrations. These three lines of evidence converge on the same conclusion: the 70:30 blend ratio combines intrinsically favorable phase structure with process-compatible morphology, which collectively underpin its optimal dimensional accuracy.

### 3.3. Orthogonal Experiment

#### 3.3.1. Orthogonal Experiment Design

In the preliminary single-factor experiments, the effects of process parameters *A*, *B*, *C*, and *D* on the dimensional deviations of PLA/TPU composite FDM-printed parts were systematically evaluated, and the PLA/TPU mass ratio of 70:30 was identified as the optimal formulation. To further optimize the forming process under this ratio, an orthogonal array approach was employed for multi-factor process optimization. Given that factors *A* and *C* were set at four levels while *B* and *D* at three levels in the single-factor trials, a standard equal-level orthogonal table was not directly applicable. Thus, a mixed-level orthogonal design L_16_ was adopted [37]. Specifically, the standard L_16_(4^5^) orthogonal table served as the base framework, and the three-level factors *B* and *D* were accommodated using the pseudo-level method to fit the four-level column structure (denoted as 4(2), indicating that the second level of the corresponding factor was repeated). The fifth column was left unassigned to provide degrees of freedom for estimating experimental error in the ANOVA. The factor levels are presented in Table 4. Dimensional deviations in the *L*, *W*, *ID*, and *OD* directions were taken as response variables (since all deviations were negative, the larger-the-better characteristic was applied, i.e., closer to zero is preferred). A total of 16 experimental runs were carried out, each with three repetitions, and the results are compiled in Table 5. Range analysis was initially conducted to determine the relative importance of each factor and the optimal level combination. Subsequently, repeated-measures multi-factor analysis of variance (fixed factors: *A*, *B*, *C*, *D*; no interaction terms) was performed using all 48 measured values to examine the statistical significance of each factor’s effect. While range analysis offers simplicity and direct interpretability, it lacks inferential statistical support; ANOVA addresses this limitation. The complementary use of these two approaches enhances the reliability and credibility of the optimization outcomes.

#### 3.3.2. Range Analysis and Determination of Optimal Levels

For each factor and each level, the mean dimensional deviation in the four directions (*L*, *W*, *ID*, and *OD*) was calculated, with each level mean representing the arithmetic average of measurements from three parallel specimens. Since all dimensional deviations were negative, the larger-the-better characteristic was adopted for evaluation, i.e., the level with the maximum mean deviation (minimum absolute value, closest to zero) was identified as the optimal level for that factor. The results of the range analysis for each factor on the four dimensional deviation indicators are summarized in Table 6.

As shown in Table 6, the range analysis for the four dimensional deviations (*L*, *W*, ID, and *OD*) yields the order of influence of each factor and the corresponding optimal level combinations, which are summarized as follows:

⋅ *L*: Factor priority: *C* (*R* = 0.070) > *D* (0.063) > *B* (0.058) > *A* (0.019); optimal combination: *A*_4_*B*_2_*C*_4_*D*_1_.
⋅ *W*: Factor priority: *D* (*R* = 0.112) > *C* (0.102) > *B* (0.086) > *A* (0.040); optimal combination: *A*_4_*B*_2_*C*_4_*D*_1_.
⋅ *ID*: Factor priority: *A* (*R* = 0.101) > *D* (0.081) > *C* (0.058) > *B* (0.056); optimal combination: *A*_1_*B*_3_*C*_1_*D*_1_.
⋅ *OD*: Factor priority: *B* (*R* = 0.082) > *D* (0.063) > *C* (0.055) > *A* (0.022); optimal combination: *A*_3_*B*_2_*C*_1_*D*_3_.

Notably, the optimal parameter combinations differ considerably across the four dimensional responses. *L* and *W* share the same optimal combination (*A*_4_*B*_2_*C*_4_*D*_1_), both favoring high printing speed (*A*_4_), moderate printing temperature (*B*_2_), and high infill rate (*C*_4_). In contrast, *ID* favors low printing speed (*A*_1_) and relatively high printing temperature (*B*_3_), while *OD* requires low infill rate (*C*_1_) and moderate printing temperature (*B*_2_). These observations indicate that different geometric features respond distinctly to process parameters: linear dimensions (*L* and *W*) are most sensitive to infill rate *C*, whereas *ID* is primarily governed by printing speed *A*, and *OD* is most strongly influenced by printing temperature *B*. Consequently, single-response optimization fails to yield a globally optimal parameter set, necessitating a multi-objective approach for comprehensive trade-offs among the four dimensional responses.

Based on Table 7, the ANOVA results for each directional dimensional deviation are summarized as follows:

For length (*L*) deviation, the established statistical model achieves statistical significance with *p* = 0.028 and a coefficient of determination *R*^2^ = 0.440. Factor *B* (printing temperature) delivers a remarkably significant impact, with *F*(3, 35) = 3.155, *p* = 0.037 and partial *η*^2^ = 0.211; per Cohen’s standard, this partial *η*^2^ value above 0.14 corresponds to a large effect size. Factor *C* (infill rate) fails to meet the statistical significance threshold at *p* = 0.063 yet yields a large effect size of partial *η*^2^ = 0.185, which reveals an obvious underlying influence trend obscured by intrasample random residual error. Factor *D* (layer thickness) possesses a moderate-to-large effect size of partial *η*^2^ = 0.142 without statistical significance (*p* = 0.136), while Factor *A* (printing speed) only contributes a trivial effect (partial *η*^2^ = 0.040, *p* = 0.706). Although range analysis ranks infill rate (Factor *C*) as the most influential parameter for length deviation, ANOVA results prove that the measured effect of *C* is severely interfered with by within-group random residuals, whereas printing temperature (Factor *B*) exerts a stable, statistically reliable influence.

In terms of width (*W*) deviation, the regression model is highly significant (*p* < 0.001, *R*^2^ = 0.651). Factor *C* (infill rate), Factor *B* (printing temperature) and Factor *D* (layer thickness) all exhibit statistically significant or highly significant impacts (*p* ≤ 0.006), accompanied by large effect sizes of partial *η*^2^ = 0.390, 0.378 and 0.294, respectively. Factor *A* (printing speed) presents no significant effect (*p* = 0.290) with a medium effect size (partial *η*^2^ = 0.101). The factor ranking obtained from range analysis prioritizes *D*, *C* and *B*, which shows high consistency with the statistical conclusions from ANOVA.

Regarding inner diameter (*ID*) deviation, the overall model is statistically significant (*p* = 0.001, *R*^2^ = 0.573). Only Factor *A* (printing speed) reaches the highly significant level (*p* < 0.001) with a large effect size (partial *η*^2^ = 0.387), which verifies printing speed as the dominant parameter governing inner dimensional precision. Factor *B* (printing temperature) holds a large effect size (partial *η*^2^ = 0.153) yet lacks statistical significance (*p* = 0.119); Factor *C* (infill rate) delivers a medium-to-large effect (partial *η*^2^ = 0.126, *p* = 0.194), and Factor *D* (layer thickness) displays a medium effect (partial η^2^ = 0.098, *p* = 0.298). The moderate or large effect sizes of these three factors without significant *p*-values imply that their actual influences are masked by random experimental residuals under current test conditions. Range analysis consistently identifies printing speed (Factor *A*) as the primary control factor, creating strong consistency with ANOVA outputs.

For outer diameter (*OD*) deviation, the fitted model is significant (*p* = 0.001, *R*^2^ = 0.568). Factor *B* (printing temperature), Factor *D* (layer thickness) and Factor *C* (infill rate) all achieve statistical significance (*p* ≤ 0.028) and carry large effect sizes, with partial *η*^2^ equal to 0.297, 0.287 and 0.192 separately. Factor *A* (printing speed) has no significant influence (*p* = 0.646) and only a small effect size (partial *η*^2^ = 0.040). The factor importance ranking generated by range analysis places *B*, *D* and *C* as the top three parameters, perfectly matching the statistical findings from ANOVA.

Collectively, the factor influence rankings derived from range analysis and ANOVA are highly consistent for width, inner diameter and outer diameter deviations. Nevertheless, an obvious discrepancy emerges for length deviation: range analysis identifies infill rate (Factor *C*) as the leading factor, while ANOVA only confirms printing temperature (Factor *B*) to be statistically significant, with infill rate falling short of the significance cutoff. This contrast indicates that range analysis can rapidly screen parameters with apparent magnitude of influence yet cannot distinguish whether such observed trends stem from intrinsic parameter effects or random residual noise. In comparison, ANOVA supported by repeated measurement data enables more rigorous statistical judgment of parameter reliability. The combination of range analysis and ANOVA therefore delivers complementary advantages: it enables fast sorting of primary and secondary influencing factors while conducting strict statistical verification of their significance, which forms a robust analytical workflow for multi-index optimization based on orthogonal experiments.

#### 3.3.3. Explanatory Power of the ANOVA Models

The *R*^2^ values for the *L*, *W*, *ID*, and *OD* directional models in this study are 0.440, 0.651, 0.573, and 0.568, respectively. The *W*-direction model exhibits a relatively high explanatory power (*R*^2^ = 0.651), while the *R*^2^ values for the *L*, *ID*, and *OD* directions fall within a moderate range. This phenomenon is commonly observed in main-effect-only models, as the FDM printing process involves coupled multi-physics fields, including melt rheology, heat transfer, and crystallization kinetics, which cannot be fully captured by a main-effect model alone [39]. Critically, all four models are statistically significant (*p* < 0.05), and the results derived from ANOVA are consistent with those from the range analysis, mutually validating the findings. Therefore, the statistical conclusions of this study are considered reliable. To further enhance the explanatory power of the model, future work may adopt a full-factorial experimental design incorporating interaction terms such as *B* × *C* and *A* × *B* [40].

### 3.4. Multi-Indicator Comprehensive Evaluation and Range Optimization Based on the Entropy Weight Method

#### 3.4.1. Determination of Indicator Weights Using the Entropy Weight Method

Based on the mean dimensional deviations of *L*, *W*, *ID*, and *OD* from the 16 experimental runs in Table 5 (averages of three parallel specimens per run), the entropy weight method was employed to determine the objective weight of each indicator, thereby eliminating the influence of subjective assignment on the comprehensive evaluation. The results show that the weights of *L*, *W*, *ID*, and *OD* are 0.209, 0.342, 0.364, and 0.084, respectively. Among these, the *ID* deviation possesses the largest weight (0.364), indicating that the inner diameter contributes most significantly to the overall dimensional accuracy and serves as a critical indicator affecting the forming quality. In contrast, the *OD* deviation has the smallest weight (0.084), suggesting a relatively limited contribution. This weight distribution reflects the differential sensitivity of various geometric features to process parameter fluctuations during FDM printing, providing an objective basis for the subsequent comprehensive scoring and range optimization.

#### 3.4.2. Range Analysis of Comprehensive Scores and Multi-Objective Optimization

Based on the weights determined by the entropy weight method ($w_{L}=0.209,w_{W}=0.342,w_{ID}=0.364,w_{OD}=0.084$), the comprehensive score *Y* for each experimental run was calculated using Equation (2),

(2) $$Y=w_{L}\times L+w_{W}\times W+w_{ID}\times ID+w_{OD}\times OD$$

following the smaller-the-better characteristic, where a smaller *Y* value indicates better overall dimensional accuracy. The resulting *Y* values are listed in the 10th column of Table 5. Range analysis was then performed using the comprehensive score *Y* as the response variable, and the results are presented in Table 8.

Based on Table 8, the factors affecting the comprehensive score *Y* are ranked in the following order of significance: *C* (*R* = 0.449) > *D* (0.298) > *B* (0.277) > *A* (0.246). Since *Y* follows the smaller-the-better characteristic, the optimal level for each factor corresponds to the smallest k value, yielding the combination *A*_4_*B*_3_*C*_4_*D*_1_, i.e., printing speed at level 4 (*A*_4_), printing temperature at level 3 (*B*_3_), infill rate at level 4 (*C*_4_), and printing distance at level 1 (*D*_1_).

This globally optimal combination (*A*_4_*B*_3_*C*_4_*D*_1_) differs from the optimal combinations identified for individual directional deviations. Specifically, the optimal combinations for the *L*- and *W*-directions are *A*_4_*B*_2_*C*_4_*D*_1_, for the *ID*-direction *A*_1_*B*_3_*C*_4_*D*_1_, and for the *OD*-direction *A*_3_*B*_2_*C*_1_*D*_3_. The globally optimal combination adopts *B*_3_ (instead of *B*_2_ favored by the W-direction) and *C*_4_ (consistent with the *L*-, *W*-, and *ID*-directions, but different from the *OD*-direction), reflecting a trade-off inherent in multi-objective optimization. The entropy weight method assigns the highest weight to *ID* deviation (0.364), followed by *W* deviation (0.342), indicating that the comprehensive score is predominantly governed by these two dimensional responses. Range analysis for *ID* recommends *C*_4_ and *B*_3_, while that for W recommends *C*_4_ and *B*_2_. The global optimum adopts *C*_4_, which is consistently recommended by both *ID* and *W*, and favors *B*_3_ as recommended by *ID*, which carries a higher weight. Accordingly, this combination achieves the best overall dimensional accuracy while prioritizing the major directional deviations, and can thus be recommended as the optimal process parameter set for multi-objective optimization.

### 3.5. Engineering Application Recommendations

Based on the above single-response and multi-objective analyses, the recommended process parameters for different engineering requirements are presented in Table 9.

It should be noted that the recommended parameters in Table 9 were obtained on a specific printer with a 0.4 mm nozzle and fixed slicer. While the absolute optimal values may shift when transferring these settings to different printers, nozzle diameters, or slicing software, the dominance ranking—particularly the primary roles of infill rate and layer height—is expected to remain consistent across similar FDM platforms. Likewise, these parameters were established on a specific specimen geometry; while this geometry captures fundamental dimensional characteristics, complex geometries may exhibit different cooling gradients and residual stress distributions, potentially requiring parameter adjustments. Accordingly, the parameter sets in Table 9 serve as starting references, and users are advised to conduct small-scale calibration experiments when applying them to different equipment or part geometries.

### 3.6. Experimental Validation

To verify the reliability of the optimization results, specimens were reprinted using the recommended parameter combinations listed in Table 9, with each combination repeated three times. The dimensional deviations in each direction were measured, and the means and standard deviations were calculated. The results are presented in Table 10.

Based on Table 10, the group prioritizing balanced multi-directional accuracy (*A*_1_*B*_3_*C*_4_*D*_1_) exhibits a total absolute deviation of 0.256 mm, which is substantially lower than those of the single-objective optimization groups (ranging from 0.321 to 0.418 mm), indicating the best overall dimensional accuracy among all strategies. Moreover, the standard deviations of this group across all directions range from 0.007 to 0.013 mm, demonstrating small and isotropic fluctuations, which confirms that this parameter combination offers good repeatability and process stability. In contrast, although the *L/W*-priority group achieves the smallest deviations in the *L* and *W* directions, it shows a relatively large standard deviation in *OD* deviation (0.022 mm), suggesting poor reproducibility of the outer diameter. Similar issues are observed in the *ID*-priority and *OD*-priority groups, each performing well only in their respective prioritized directions. These comparisons further indicate that single-objective optimization fails to simultaneously satisfy all directional accuracy requirements, whereas the balanced multi-directional group, through the entropy weight-TOPSIS multi-objective optimization, attains a comprehensive trade-off among all directional dimensions. The close agreement between the validation results and the optimization predictions demonstrates the reliability and engineering practicality of the multi-objective optimization methodology adopted in this study.

## 4. Discussion

### 4.1. Influence of Blend Ratio on Dimensional Accuracy and Underlying Mechanisms

The single-factor experimental results demonstrate that dimensional accuracy improves monotonically with increasing PLA content, following the ranking: 90:10 > 85:15 > 80:20 > 75:25 > 70:30. This trend can be explained by the higher rigidity and lower coefficient of thermal expansion of PLA compared with TPU. As the TPU proportion increases, the elastomeric phase introduces greater thermal shrinkage and viscoelastic relaxation upon cooling, both of which amplify the final dimensional deviations [35].

Interestingly, the TOPSIS comprehensive evaluation identifies the 70:30 ratio as the optimal composition (relative closeness = 0.680), despite it exhibiting the largest individual deviations among all ratios in the single-factor tests. This apparent paradox is resolved by the multi-dimensional nature of the evaluation: the 70:30 composition achieves the most balanced performance across the four measured features (*L*, *W*, *ID*, and *OD*). In contrast, ratios with higher PLA content may excel in specific dimensions but perform poorly in others, resulting in lower overall comprehensive scores.

The mechanistic origin of this balanced performance is supported by both XRD and SEM analyses. XRD reveals that the 70:30 filaments exhibit the lowest crystallinity (12.3%) among all blends, coinciding with a phase inversion from PLA-continuous to TPU-continuous morphology. This substantial reduction in crystallinity implies a narrower volume change during solidification, which helps mitigate non-uniform shrinkage. The observed non-monotonic crystallinity dependence on TPU content, where low TPU fractions (90:10) slightly promote PLA crystallization via heterogeneous nucleation (increasing from 18.2% to 23.1%) [33], while higher TPU fractions progressively suppress it down to 12.3%, is consistent with the spatial hindrance mechanism reported in PLA/TPU blend systems [32].

SEM further confirms that the 70:30 printed parts, when processed under blend-matched parameters, achieve the finest and most homogeneous dispersion of TPU domains, together with the best interfacial adhesion and minimal void formation. This morphological excellence ensures uniform stress distribution during cooling and eliminates localized strain concentrations that would otherwise amplify dimensional deviations. In contrast, the coarse and aggregated TPU domains and poor interfacial bonding observed under PLA-optimized conditions cause non-uniform melt rheology and localized stress concentrations during solidification, thereby exacerbating dimensional deviations. The low crystallinity of the 70:30 filaments ensures minimal and more uniform solidification shrinkage during printing, while the fine and well-bonded morphology of the printed parts guarantees homogeneous stress distribution throughout cooling.

### 4.2. Feature-Dependent Parameter Sensitivity

The orthogonal experiments and ANOVA reveal a clear hierarchy of parameter importance that varies across geometric features. Infill rate (*C*) is the dominant factor for *L*- and *W*-deviations, while *ID*-deviation is primarily controlled by printing speed (*A*), and *OD*-deviation is most sensitive to printing temperature (*B*). This feature-specific response pattern indicates that different geometric features are governed by distinct physical mechanisms during the FDM process.

For *L* and *W* (straight features): Infill rate directly determines the internal skeleton stiffness and resistance to warping along planar directions. Higher infill rate provides greater internal support, restricting shrinkage along straight edges. The ANOVA confirms the statistical significance of infill rate for *W*-deviation (*p* < 0.001) and its primary influence on *L*-deviation through range analysis, corroborating this mechanistic interpretation.

For *ID* (circular hole): The inner diameter represents a curved boundary sensitive to extrusion flow rate. Printing speed affects the pressure buildup and residence time of molten material at corners. At higher speeds, reduced pressure may lead to insufficient filling of curved contours, while at lower speeds, excess material may cause bulging. This sensitivity explains why printing speed (*A*) is the most significant factor for *ID*-deviation (*p* < 0.001).

For *OD* (outer circular contour): The outer diameter is exposed to ambient convection on one side and retains heat from the adjacent interior on the other, creating a thermal gradient across the section. The outer contour also suffers from arc-fitting toolpath errors due to the conversion of curved surfaces into segmented linear movements in G-code [41,42]. These two effects—uneven edge cooling and cumulative trajectory errors—are inherently temperature-dependent. This explains why printing temperature (B) exerts the strongest influence on *OD*-deviation (*p* = 0.002), with layer height (*D*, *p* = 0.002) and infill rate (*C*, *p* = 0.028) also showing statistical significance.

The consistency between range analysis and ANOVA across all features provides a reliable statistical basis for the multi-response optimization conducted in this study.

### 4.3. Implications for Precision FDM Manufacturing

The entropy weight method assigns the largest weight to *ID*-deviation (0.364), followed by *W*-deviation (0.342), with *L*- (0.209) and *OD*-deviations (0.084) contributing relatively less. This weight distribution indicates that, within the present experimental range, the inner diameter exhibits the highest sensitivity to process variations, while the outer diameter shows the greatest robustness.

The validation experiments confirm that the balanced parameter set (*A*_4_*B*_3_*C*_4_*D*_1_: 60 mm/s, 210 °C, 90% infill, 0.1 mm layer height) achieves a total absolute deviation of 0.256 mm, markedly lower than those of the single-objective optimization groups (0.321–0.418 mm), with standard deviations of 0.007–0.013 mm across all directions. These values indicate good repeatability and process stability under the optimized conditions.

The close agreement between the validation results and the optimization predictions demonstrates that the entropy weight–TOPSIS coupled model can effectively balance competing dimensional requirements. The four recommended parameter sets (*L/W*-priority, *ID*-priority, *OD*-priority, and balanced multi-directional) provide practical flexibility for different engineering applications. For example, components requiring tight internal fit (e.g., bearing housings) should prioritize *ID* accuracy, whereas parts with critical planar contact surfaces should emphasize *W*-accuracy. This application-tailored approach enhances the practical utility of the optimization framework beyond the specific material system investigated.

### 4.4. Limitations and Future Work

We acknowledge several limitations of this study. First, the investigation is confined to the PLA/TPU blend system; the applicability of the optimization framework to other polymer blends requires further validation. Second, dimensional accuracy was assessed only at room temperature immediately after printing; the thermal stability of dimensions under elevated temperatures or cyclic loading was not examined. Third, the optimized parameters were obtained on a single printer platform with a 0.4 mm nozzle and fixed slicer, and using a specific specimen geometry; their generalizability to other equipment configurations or part geometries requires further validation. Fourth, the orthogonal array employed in this study primarily captures main effects without estimating parameter interactions, which will be systematically addressed in future response surface work. Fifth, while the microstructure–accuracy correlation is established through XRD and SEM observations, direct in situ verification (e.g., real-time thermal imaging during printing or strain mapping) was not performed. The interpretations regarding melt flow behavior are indirectly inferred from observed dimensional trends, as dedicated rheological characterization was not performed. Additionally, complementary techniques such as differential scanning calorimetry (DSC), Fourier-transform infrared spectroscopy (FTIR), or dynamic mechanical analysis (DMA) were not employed, which would have provided further evidence of blend compatibility through glass transition behavior, intermolecular interactions, and viscoelastic properties. Future work will incorporate these characterization methods to establish a more comprehensive understanding of the structure–property relationship in PLA/TPU blends.

Future work will extend the current framework to incorporate mechanical properties—such as tensile strength, stiffness, and impact resistance—as additional optimization objectives, enabling a more comprehensive material and process design. Beyond mechanical performance, the framework could be extended to other material systems and applied to assess long-term dimensional stability under environmental aging (humidity, temperature cycling). Additionally, integrating machine learning with the entropy weight–TOPSIS framework could enable adaptive parameter tuning based on real-time sensor feedback, paving the way for closed-loop precision control in FDM. These investigations will contribute to the broader goal of transforming FDM from a prototyping tool into a reliable production technology for high-accuracy functional parts.

### 4.5. Comparison with Recent PLA/TPU FDM Optimization Studies

To contextualize the findings of this study, Table 11 compares the key results with the recent literature on PLA/TPU FDM optimization.

The comparison reveals several important observations. First, the optimal blend ratio identified in this study (70:30) is consistent with Rahmatabadi et al. [15], who also employed a 70:30 PLA/TPU composition for FDM printing. Second, regarding parameter significance, our finding that infill rate and layer height are the dominant factors aligns with the general trend in the literature [9,15,24], while the relatively minor role of printing temperature corroborates the observations of Rahmatabadi et al. [15], who reported nozzle temperature as the least influential parameter. Third, while most existing studies focus on mechanical or shape memory properties [7,9,13,15], the present work specifically addresses dimensional accuracy—a critical but less explored aspect of PLA/TPU FDM printing—and establishes a systematic multi-objective optimization framework integrating single-factor experiments, orthogonal design, and entropy weight–TOPSIS. This methodological combination has not been previously reported for dimensional accuracy optimization of PLA/TPU blends, distinguishing the present work from the existing literature.

## 5. Conclusions

This study systematically investigated the coupled effects of PLA/TPU blend ratio and FDM process parameters on dimensional accuracy through an integrated approach combining orthogonal array design, ANOVA, and entropy weight–TOPSIS multi-objective optimization. The main conclusions are as follows:

(1) The PLA/TPU blend ratio significantly affects the dimensional accuracy of FDM-printed parts. Accuracy improves monotonically with increasing PLA content, following the order: 90:10 > 85:15 > 80:20 > 75:25 > 70:30, which is attributed to PLA’s higher rigidity and lower thermal shrinkage relative to TPU.
(2) Despite ranking lowest in single-factor accuracy, the 70:30 blend achieves the highest TOPSIS relative closeness (0.680), identifying it as the optimal composition. XRD reveals its lowest crystallinity (12.3%) among all blends, while SEM confirms the finest, most homogeneous TPU dispersion with the best interfacial adhesion. These complementary features—minimal solidification shrinkage and uniform stress distribution—jointly underpin its superior balanced dimensional accuracy.
(3) Range analysis and ANOVA consistently demonstrate that parameter dominance is feature-dependent: Infill rate (*C*) governs *L*- and *W*-deviations, printing speed (*A*) controls *ID*-deviation, and printing temperature (*B*) predominantly affects *OD*-deviation. This feature-specific sensitivity confirms that no single parameter set can universally optimize all dimensions, highlighting the necessity of multi-response optimization.
(4) Entropy weights rank *ID*-deviation highest (0.364), followed by *W*- (0.342), *L*- (0.209), and *OD*-deviation (0.084). The globally optimal parameter combination is *A*_4_*B*_3_*C*_4_*D*_1_ (printing speed: 60 mm/s, printing temperature: 210 °C, infill rate: 90%, layer height: 0.1 mm). Four application-tailored parameter sets are provided to accommodate varying engineering priorities.
(5) Validation confirms that the balanced optimized group (*A*_4_*B*_3_*C*_4_*D*_1_) achieves a total absolute deviation of 0.256 mm, substantially lower than single-objective groups (0.321–0.418 mm), with standard deviations of 0.007–0.013 mm across all directions, demonstrating excellent reproducibility. The strong agreement between validation and predictions confirms the reliability and practical value of the entropy weight–TOPSIS framework, offering both mechanistic insights and actionable guidelines for high-precision FDM manufacturing of polymer blends.

## Figures and Tables

**Table 1:** Single-Factor Variable Experiment Parameter Settings.

Items	Factor			
	*A* (mm/s)	*B* (°C)	*C* (%)	*D* (mm)
Min	30	190	60	0.1
Max	60	210	90	0.3
Interval	10	10	10	0.1
Fixed value	40	200	80	0.2

**Table 2:** Optimal process parameters for each material system.

Material System	Optimal Process Parameters (*A*/*B*/*C*/*D*)	*L* (mm)	*W* (mm)	*ID* (mm)	*OD* (mm)
Neat PLA	30 mm/s; 210 °C; 90%; 0.3 mm	−0.103 ± 0.008	−0.014 ± 0.002	−0.099 ± 0.009	−0.087 ± 0.005
90:10	50 mm/s; 200 °C; 60%; 0.3 mm	−0.240 ± 0.001	−0.201 ± 0.003	−0.056 ± 0.001	−0.172 ± 0.005
85:15	30 mm/s; 190 °C; 90%; 0.3 mm	−0.223 ± 0.002	−0.114 ± 0.001	−0.218 ± 0.002	−0.193 ± 0.001
80:20	60 mm/s; 210 °C; 90%; 0.3 mm	−0.164 ± 0.001	−0.047 ± 0.001	−0.251 ± 0.002	−0.031 ± 0.001
75:25	60 mm/s; 190 °C; 80%; 0.2 mm	−0.080 ± 0.001	−0.126 ± 0.001	−0.147 ± 0.001	−0.233 ± 0.001
70:30	50 mm/s; 210 °C; 60%; 0.3 mm	−0.271 ± 0.001	−0.188 ± 0.001	−0.087 ± 0.001	−0.231 ± 0.002

Note: Mean ± SD (*n* = 3). Negative values denote shrinkage.

**Table 3:** Comprehensive evaluation results and ranking of each material system based on the entropy weight–TOPSIS method.

**Material System**	$D_{i}^{+}$	$D_{i}^{-}$	$C_{i}$
**Neat PLA**	0.300	0.061	0.169
**90:10**	0.262	0.357	0.577
**85:15**	0.194	0.336	0.634
**80:20**	0.440	0.238	0.352
**75:25**	0.231	0.326	0.586
**70:30**	0.202	0.428	0.680

Note: The indicator weights (*L*/*W*/*ID*/*OD*) were determined by the entropy weight method as 0.236, 0.232, 0.292, and 0.240, respectively. $D_{i}^{+}$ is the weighted Euclidean distance from the positive ideal solution (the optimal value of each indicator)—a smaller value indicates closer proximity to the ideal forming quality. $D_{i}^{-}$ is the weighted Euclidean distance from the negative ideal solution (the worst value of each indicator)—a larger value indicates greater deviation from the poorest forming state. The relative closeness $C_{i}=\frac{D_{i}^{-}}{D_{i}^{+}+D_{i}^{-}}$ serves as the comprehensive evaluation index, where a larger $C_{i}$ value denotes better overall dimensional accuracy. The blend ratios are ranked in descending order of $C_{i}$.

**Table 4:** Factor levels for the orthogonal experimental design.

Level	Factor			
	*A*/(mm/s)	*B* (°C)	*C* (%)	*D* (mm)
1	30	190	60	0.1
2	40	200	70	0.2
3	50	210	80	0.3
4 ^1^	60	200 *	90	0.2 *

* Note: Pseudo-level, reusing the Level 2 parameter.

**Table 5:** Orthogonal experimental design and results.

Run	*A* (mm/s)	*B* (°C)	*C* (%)	*D* (mm)	*L* (mm)	*W* (mm)	*ID* (mm)	*OD* (mm)	*Y* ^1^
1	1	1	1	1	−0.102	−0.039	−0.040	−0.180	0.106
2	1	2	2	2	−0.194	−0.144	−0.060	−0.211	0.432
3	1	3	3	3	−0.152	−0.111	−0.058	−0.164	0.311
4	1	4	4	4	−0.169	−0.121	−0.044	−0.227	0.335
5	2	1	2	3	−0.187	−0.143	−0.167	−0.173	0.663
6	2	2	1	4	−0.076	−0.032	−0.187	−0.067	0.374
7	2	3	4	1	−0.079	−0.035	−0.076	−0.180	0.156
8	2	4	3	2	−0.203	−0.273	−0.179	−0.321	0.946
9	3	1	3	4	−0.230	−0.172	−0.145	−0.184	0.711
10	3	2	4	1	−0.069	−0.034	−0.056	−0.124	0.075
11	3	3	1	2	−0.188	−0.138	−0.060	−0.210	0.416
12	3	4	2	3	−0.126	−0.092	−0.138	−0.187	0.448
13	4	1	4	2	−0.118	−0.044	−0.033	−0.197	0.124
14	4	2	3	3	−0.129	−0.085	−0.167	−0.208	0.518
15	4	3	2	4	−0.090	−0.043	−0.051	−0.188	0.125
16	4	4	1	1	−0.202	−0.152	−0.033	−0.200	0.389

^1^ Note: *Y* denotes the weighted comprehensive score of the four-dimensional deviations (weights determined by the entropy weight method), following the smaller-the-better characteristic, where a smaller *Y* value indicates better overall dimensional accuracy. The specific calculation formula is provided in Section 3.4.2.

**Table 6:** Range analysis results for dimensional deviations.

Response	Statistic	*A* (mm/s)	*B* (°C)	*C* (%)	*D* (mm)
*L*	*k*_1_/*k*_2_/ *k*_3_/*k*_4_	−0.154/−0.136/ −0.153/−0.135	−0.159/−0.117/ −0.127/−0.175	−0.142/−0.149/ −0.179/−0.109	−0.113/−0.176/ −0.149/−0.141
	*R*	0.019	0.058	0.070	0.063
*W*	*k*_1_/*k*_2_/ *k*_3_/*k*_4_	−0.104/−0.121/ −0.109/−0.081	−0.100/−0.074/ −0.082/−0.160	−0.090/−0.106/ −0.160/−0.058	−0.038/−0.150/ −0.108/−0.092
	*R*	0.040	0.086	0.102	0.112
*ID*	*k*_1_/*k*_2_/ *k*_3_/*k*_4_	−0.051/−0.152/ −0.100/−0.071	−0.096/−0.117/ −0.061/−0.099	−0.080/−0.104/ −0.137/−0.079	−0.051/−0.083/ −0.132/−0.107
	*R*	0.101	0.056	0.058	0.081
*OD*	*k*_1_/*k*_2_/ *k*_3_/*k*_4_	−0.196/−0.185/ −0.176/−0.198	−0.184/−0.152/ −0.186/−0.234	−0.164/−0.190/ −0.219/−0.182	−0.171/−0.235/ −0.183/−0.172
	*R*	0.022	0.082	0.055	0.063

Note: *k*_1_–*k*_4_ represent the mean deviations at levels 1–4 for each factor; *R* is the range, i.e., the difference between the maximum and minimum mean deviations among the levels of each factor.

**Table 7:** ANOVA results for dimensional deviations in the *L*, *W*, *ID*, and *OD* directions.

Response	Source of Variation	Sum of Squares	Degree of Freedom	Mean Square	*F*-Value	*p*-Value ^1^	Partial *η*^2^
*L*	Model	0.076	12	0.006	2.294	0.028	
	*A*	0.004	3	0.001	0.469	0.706	0.040
	*B*	0.026	3	0.009	3.155	0.037	0.211
	*C*	0.022	3	0.007	2.669	0.063	0.185
	*D*	0.016	3	0.005	1.975	0.136	0.142
	Residual	0.097	35	0.003			
*W*	Model	0.166	12	0.014	5.438	<0.001	
	*A*	0.010	3	0.003	1.299	0.290	0.101
	*B*	0.054	3	0.018	7.058	0.001	0.378
	*C*	0.057	3	0.019	7.476	0.001	0.390
	*D*	0.037	3	0.012	4.853	0.006	0.294
	Residual	0.089	35	0.003			
*ID*	Model	0.148	12	0.012	3.908	0.001	
	*A*	0.070	3	0.023	7.348	0.001	0.387
	*B*	0.020	3	0.007	2.094	0.119	0.153
	*C*	0.016	3	0.005	1.657	0.194	0.126
	*D*	0.012	3	0.004	1.276	0.298	0.098
	Residual	0.111	35	0.003			
*OD*	Model	0.102	12	0.009	3.843	0.001	
	*A*	0.004	3	0.001	0.558	0.646	0.040
	*B*	0.041	3	0.014	6.109	0.002	0.297
	*C*	0.023	3	0.008	3.406	0.028	0.192
	*D*	0.039	3	0.013	5.832	0.002	0.287
	Residual	0.097	35	0.005			

^1^ Note: *p* < 0.05 indicates statistical significance; *p* < 0.01 indicates high significance. *p*-values less than 0.001 are reported as “<0.001”. Partial *η*^2^ values represent effect sizes, with values of 0.01, 0.06, and 0.14 indicating small, medium, and large effects, respectively [38].

**Table 8:** Range analysis results for the comprehensive score Y.

Level	*A* (mm/s)	*B* (°C)	*C* (%)	*D* (mm)
*K*_1_	0.296	0.401	0.321	0.181
*K*_2_	0.535	0.350	0.417	0.479
*K*_3_	0.412	0.252	0.621	0.485
*K*_4_	0.289	0.529	0.172	0.386
*R*	0.246	0.277	0.449	0.298
Optimal level	*A*_4_ *B*_3_ *C*_4_ *D*_1_			

Note: *K*_1_–*K*_4_ represent the mean comprehensive scores at levels 1–4 for each factor; *R* is the range, i.e., the difference between the maximum and minimum mean comprehensive scores among the levels of each factor.

**Table 9:** Recommended process parameters for different engineering requirements.

Optimization Objective	Recommended Combination	Basis
Priority on L/W accuracy	*A*_4_ *B*_2_ *C*_4_ *D*_1_	Optimal combination from *L*/*W* range analysis
Priority on ID accuracy	*A*_1_ *B*_3_ *C*_4_ *D*_1_	Largest *R* for A in *ID* range analysis; *A* is highly significant in ANOVA
Priority on OD accuracy	*A*_3_ *B*_2_ *C*_1_ *D*_3_	Optimal combination from *OD* range analysis
Balanced multi-directional accuracy	*A*_4_ *B*_3_ *C*_4_ *D*_1_	Range analysis of comprehensive score *Y* by entropy weight method (smaller-the-better)

Note: *A*_4_*B*_2_*C*_4_*D*_1_ and *A*_4_*B*_3_*C*_4_*D*_1_ share the same *A*_4_ and *C*_4_ levels, differing primarily in printing temperature (*B*_2_ vs. *B*_3_), indicating that temperature adjustment serves as the critical parameter when balancing multi-directional accuracy requirements. Users may fine-tune between *B*_2_ and *B*_3_ according to the specific accuracy requirements of *L*/*W* and *ID* directions in practical applications.

**Table 10:** Validation results for different optimization objectives (mean ± SD).

Optimization Objective	Recommended Combination	*L* (mm)	*W* (mm)	*ID* (mm)	*OD* (mm)	Total Absolute Deviation
Priority on L/W accuracy	*A*_4_ *B*_2_ *C*_4_ *D*_1_	−0.018 ± 0.006	−0.035 ± 0.008	−0.112 ± 0.015	−0.156 ± 0.022	0.321
Priority on ID accuracy	*A*_1_ *B*_3_ *C*_4_ *D*_1_	−0.087 ± 0.012	−0.052 ± 0.010	−0.042 ± 0.005	−0.203 ± 0.028	0.384
Priority on OD accuracy	*A*_3_ *B*_2_ *C*_1_ *D*_3_	−0.126 ± 0.018	−0.098 ± 0.014	−0.156 ± 0.020	−0.038 ± 0.004	0.418
Balanced multi-directional accuracy	*A*_4_ *B*_3_ *C*_4_ *D*_1_	−0.024 ± 0.007	−0.041 ± 0.009	−0.089 ± 0.011	−0.102 ± 0.013	0.256

Note: Data are presented as mean ± SD (*n* = 3). Total absolute deviation is calculated as |*L*| + |*W*| + |*ID*| + |*OD*| for overall comparison of dimensional accuracy among different optimization objectives.

**Table 11:** Comparison of key findings with recent PLA/TPU FDM optimization studies.

Study	PLA/TPU Ratio	Optimization Method	Key Findings
Rahmatabadi et al. (2023) [15]	70:30	BBD	Infill density most influential; nozzle temperature least influential
Hamidi et al. (2025) [13]	Multiple	Single-factor + ANOVA	TPU content and printing parameters jointly affect thermal/mechanical properties
Yurtbasi et al. (2025) [11]	PLA/TPU blends	Rheological + mechanical	TPU(A60) improves flexibility but compromises dimensional accuracy
Goh et al. (2025) [14]	75:25	Multi-material FGF	Toughness peaks at 75:25; interface shear strength +320%
Wang & Tang (2025) [24]	PLA	Orthogonal + Entropy TOPSIS	Layer height and nozzle temperature dominate dimensional accuracy
**This study**	70:30	Single-factor + Orthogonal + Entropy TOPSIS	70:30 optimal for dimensional accuracy; infill rate and layer height dominant (*p* < 0.05); cumulative deviation 0.256 mm

## Data Availability

The raw data supporting the conclusions of this article will be made available by the authors on request.

## Abbreviations

The following abbreviations are used in this manuscript:

PLA	Polylactic acid
TPU	Thermoplastic polyurethane
FDM	Fused deposition modeling
XRD	X-ray diffraction
SEM	Scanning electron microscopy
ANOVA	Analysis of variance
TOPSIS	Technique for Order Preference by Similarity to Ideal Solution
*L*	Length deviation
*W*	Width deviation
*ID*	Inner diameter deviation
*OD*	Outer diameter deviation
